# miR-22 has a potent anti-tumour role with therapeutic potential in acute myeloid leukaemia

**DOI:** 10.1038/ncomms11452

**Published:** 2016-04-26

**Authors:** Xi Jiang, Chao Hu, Stephen Arnovitz, Jason Bugno, Miao Yu, Zhixiang Zuo, Ping Chen, Hao Huang, Bryan Ulrich, Sandeep Gurbuxani, Hengyou Weng, Jennifer Strong, Yungui Wang, Yuanyuan Li, Justin Salat, Shenglai Li, Abdel G. Elkahloun, Yang Yang, Mary Beth Neilly, Richard A. Larson, Michelle M. Le Beau, Tobias Herold, Stefan K. Bohlander, Paul P. Liu, Jiwang Zhang, Zejuan Li, Chuan He, Jie Jin, Seungpyo Hong, Jianjun Chen

**Affiliations:** 1 Department of Cancer Biology, University of Cincinnati, Cincinnati, Ohio 45219, USA; 2 Section of Hematology/Oncology, Department of Medicine, University of Chicago, Chicago, Illinois 60637, USA; 3 Department of Hematology, The First Affiliated Hospital Zhejiang University, Hangzhou, 310003 Zhejiang, China; 4 Department of Biopharmaceutical Sciences College of Pharmacy, The University of Illinois, Chicago, Illinois 60612, USA; 5 Department of Chemistry and Institute for Biophysical Dynamics, Howard Hughes Medical Institute, University of Chicago, Chicago, Illinois 60637, USA; 6 State Key Laboratory of Oncology in South China, Collaborative Innovation Center for Cancer Medicine, Sun Yat-sen University Cancer Center, 510060 Guangzhou, China; 7 Department of Pathology, University of Chicago, Chicago, Illinois 60637, USA; 8 Division of Intramural Research, National Human Genome Research Institute, NIH, Bethesda, Maryland 20892, USA; 9 Department of Internal Medicine 3, University Hospital Grosshadern, Ludwig-Maximilians-Universität, 81377 Munich, Germany; 10 Department of Molecular Medicine and Pathology, University of Auckland, Auckland 1142, New Zealand; 11 Oncology Institute, Cardinal Bernardin Cancer Center, Loyola University Medical Center, Maywood, Illinois 60153, USA; 12 Department of Human Genetics, University of Chicago, Chicago, Illinois 60637, USA; 13 Integrated Science and Engineering Division, Underwood International College, Yonsei University, Incheon 406-840, Korea

## Abstract

MicroRNAs are subject to precise regulation and have key roles in tumorigenesis. In contrast to the oncogenic role of miR-22 reported in myelodysplastic syndrome (MDS) and breast cancer, here we show that miR-22 is an essential anti-tumour gatekeeper in *de novo* acute myeloid leukaemia (AML) where it is significantly downregulated. Forced expression of miR-22 significantly suppresses leukaemic cell viability and growth *in vitro*, and substantially inhibits leukaemia development and maintenance *in vivo*. Mechanistically, miR-22 targets multiple oncogenes, including *CRTC1*, *FLT3* and *MYCBP*, and thus represses the CREB and MYC pathways. The downregulation of miR-22 in AML is caused by TET1/GFI1/EZH2/SIN3A-mediated epigenetic repression and/or DNA copy-number loss. Furthermore, nanoparticles carrying miR-22 oligos significantly inhibit leukaemia progression *in vivo*. Together, our study uncovers a TET1/GFI1/EZH2/SIN3A/miR-22/CREB-MYC signalling circuit and thereby provides insights into epigenetic/genetic mechanisms underlying the pathogenesis of AML, and also highlights the clinical potential of miR-22-based AML therapy.

## Abstract (web summary)

Mir-22 has been shown to be an oncogenic microRNA in breast cancer and myelodysplastic syndrome. Here, the authors show that mir-22 functions as a tumour suppressor in *de novo* acute myeloid leukaemia by inhibiting the expression of several oncogenes and that restoring mir-22 expression suppresses AML progression.

## Graphical abstract

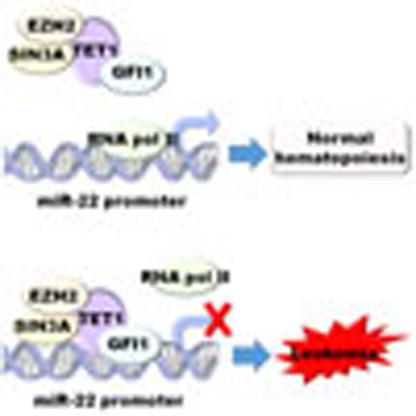

As one of the most common and fatal forms of hematopoietic malignancies, acute myeloid leukaemia (AML) is frequently associated with diverse chromosome translocations (for example t(11q23)/*MLL*-rearrangements, t(15;17)/*PML-RARA* and t(8;21)/*AML1-ETO*) and molecular abnormalities (for example, internal tandem duplications of *FLT3* (*FLT3*-ITD) and mutations in nucleophosmin (*NPM1c*^+^))^1^. Despite intensive chemotherapies, the majority of patients with AML fail to survive longer than 5 years^2^,^3^. Thus, development of effective therapeutic strategies based on a better understanding of the molecular mechanisms underlying the pathogenesis of AML is urgently needed.

MicroRNAs (miRNAs) are a class of small, non-coding RNAs that post-transcriptionally regulate gene expression^4^. Individual miRNAs may play distinct roles in cancers originating from different tissues or even from different lineages of hematopoietic cells^4^. It is unclear whether a single miRNA can play distinct roles between malignancies originating from the same hematopoietic lineage, such as *de novo* AML and myelodysplastic syndrome (MDS). Although around 30% of MDS cases transform to AML, the genetic and epigenetic landscapes of MDS or MDS-derived AML are largely different from those of *de novo* AML^5^,^6^. MDS and MDS-derived AML are more responsive to hypomethylating agents than *de novo* AML^7^. The molecular mechanisms underlying the distinct pathogenesis and drug response between MDS (or MDS-derived AML) and *de novo* AML remain unclear.

The ten-eleven translocation (Tet1/2/3) proteins play critical transcriptional regulatory roles in normal developmental processes as activators or repressors^8^,^9^,^10^. In contrast to the frequent loss-of-function mutations and tumour-suppressor role of *TET2* observed in hematopoietic malignancies^11^,^12^,^13^, we recently reported that *TET1* plays an essential oncogenic role in *MLL*-rearranged AML where it activates expression of homeobox genes^14^. However, it is unknown whether TET1 can also function as a transcriptional repressor in cancer. Moreover, Tet1-mediated regulation of miRNA expression has rarely been studied^10^.

In the present study, we demonstrate that miR-22, an oncogenic miRNA reported in breast cancer and MDS^15^,^16^, is significantly downregulated in most cases of *de novo* AML due to TET1/GFI1/EZH2/SIN3A-mediated epigenetic repression and/or DNA copy-number loss. miR-22 functions as an essential anti-tumour gatekeeper in various AML and holds great therapeutic potential to treat AML.

## Results

### The downregulation of miR-22 in *de novo* AML

**Figure 1:**
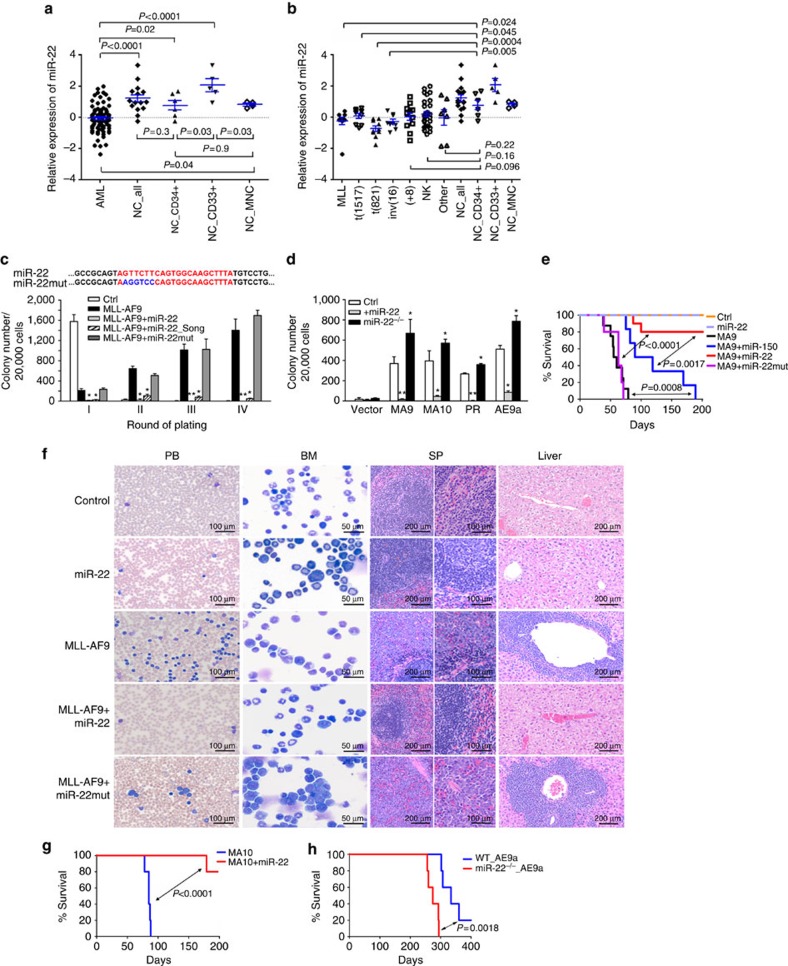
miR-22 inhibits AML cell transformation and leukemogenesis. (**a**,**b**) Exiqon microRNA profiling assay showed that miR-22 is significantly (*P*<0.05) downregulated in the entire set of AML set (*n*=85) (**a**) or each individual subset (**b**), relative to normal controls. The expression data were log(2) transformed and mean-centred. Mean±s.e.m. values were shown. (**c**) Comparison of effects of in-house miR-22, miR-22_Song^16^ and miR-22 mutant (miR-22mut; see the mutation sequence at the top) on *MLL-AF9*-induced colony forming. CFAs were performed using mouse BM progenitor (Lin^-^) cells transduced with MSCV-neo+MSCV-PIG (Ctrl), MSCV-neo-*MLL-AF9*+MSCV-PIG (MLL-AF9), or MSCV-neo-*MLL-AF9*+MSCV-PIG-miR-22/miR-22_Song/miR-22mut. (**d**) Effects of miR-22 on the colony forming induced by multiple fusion genes. CFA was performed using wild-type BM progenitor cells co-transduced with MSCV-neo-*MLL-AF9* (MA9), *-MLL-AF10* (MA10)*, -PML-RARA* (PR) or -*AML1-ETO9a* (AE9a)^19^, together with MSCV-PIG (Ctrl) or MSCV-PIG-miR-22 (+miR-22), as well as miR-22^−/−^ BM progenitors co-transduced with individual fusion genes and MSCV-PIG. Colony counts (mean±s.d.) of the second round of plating are shown. *P<0.05; ^**^P<0.01. (**e,f**) Effect of miR-22 on *MLL-AF9*-induced primary leukemogenesis. Kaplan–Meier curves are shown for six cohorts of transplanted mice including MSCVneo+MSCV-PIG (Ctrl; *n*=5), MSCVneo+MSCV-PIG-miR-22 (miR-22; *n*=5), MSCVneo-*MLL-AF9*+MSCV-PIG (MA9; *n*=8), MSCVneo-*MLL-AF9*+MSCV-PIG-miR-150 (MA9+miR-150, *n*=6), MSCVneo-*MLL-AF9*+MSCV-PIG-miR-22 (MA9+miR-22; *n*=10) and MSCVneo-*MLL-AF9*+MSCV-PIG-miR-22mutant (MA9+miR-22mut; *n*=5) (**e**); Wright–Giemsa stained PB and bone marrow (BM), and hematoxylin and eosin (H&E) stained spleen and liver of the primary BMT recipient mice at the end point are shown (**f**). (**g**) Effect of miR-22 on *MLL-AF10*-induced primary leukemogenesis. Kaplan–Meier curves are shown for two cohorts of transplanted mice including MSCVneo-*MLL-AF10*+MSCV-PIG (MA10; *n*=5) and MSCVneo-*MLL-AF10*+MSCV-PIG-miR-22 (MA10+miR-22; *n*=5). (**h**) miR-22 knockout promotes *AE9a*-induced leukemogenesis. Kaplan–Meier curves are shown for mice transplanted with wild-type or miR-22^−/−^ BM progenitor cells transduced MSCV-PIG-AE9a (*n*=5 for each group). The *P* values were generated by *t*-test (**a**–**d**) or log-rank test (**e**,**g**,**h**).

Through Exiqon miRNA array profiling, we previously identified a set of miRNAs, such as miR-150, miR-148a, miR-29a, miR-29b, miR-184, miR-342, miR-423 and miR-22, which are significantly downregulated in AML compared with normal controls^17^. Here we showed that among all the above miRNAs, miR-150 and especially miR-22 exhibited the most significant and consistent inhibitory effect on *MLL-AF9*-induced cell immortalization in colony-forming/replating assays (CFA) (Supplementary Fig. 1a). In contrast to the reported upregulation of miR-22 in MDS^16^, our original microarray data^17^ (Fig. 1a,b) and new quantitative PCR-independent validation data (Supplementary Fig. 1b) demonstrated a significant and global downregulation of miR-22 in *de novo* AML relative to normal controls. Notably, miR-22 is significantly downregulated in AML samples (*P*<0.05) compared with all three sub-populations of normal control cells, that is, normal CD34^+^ hematopoietic stem/progenitor cells (HSPCs), CD33^+^ myeloid progenitor cells, or mononuclear cells (MNCs) (Fig. 1a). Expression of miR-22 is significantly downregulated in all or the majority of individual subsets of AML samples than in the normal CD33^+^ or CD34^+^ cell samples (Fig. 1b).

To rule out the possibility that the inhibitory effect of miR-22 shown in Supplementary Fig. 1a was due to a non-specific effect of our miR-22 construct, we included the MSCV-PIG-miR-22 construct from Song *et al*.^16^ in a repeated CFA. Both miR-22 constructs dramatically inhibited *MLL-AF9*-induced colony formation (Fig. 1c). As the ‘seed’ sequences at the 5′ end of individual miRNAs are essential for the miRNA-target binding^18^, we also mutated the 6-bases ‘seed’ sequence of miR-22 and found that the miR-22 mutant did not inhibit colony formation anymore (Fig. 1c). In human AML cells, forced expression of miR-22, but not miR-22 mutant, significantly inhibited cell viability and growth/proliferation, while promoting apoptosis (Supplementary Fig. 1c,d).

Furthermore, as miR-22 is globally downregulated in all major types of AML (Fig. 1b), we also investigated the role of miR-22 in colony formation induced by other oncogenic fusion genes, including *MLL-AF10*/t(10;11), *PML-RARA*/t(15;17) and *AML1-ETO9a*/t(8;21) (ref. 19). As expected, forced expression of miR-22 significantly inhibited colony formation induced by all individual oncogenic fusions; conversely, miR-22 knockout^20^ significantly enhanced colony forming (Fig. 1d). These results suggest that miR-22 likely plays a broad anti-tumour role in AML.

In accordance with the potential anti-tumour function of miR-22 in AML, miR-22 was expressed at a significantly higher level (*P*<0.05) in human normal CD33^+^ myeloid progenitor cells than in more immature CD34^+^ HSPCs or MNC cells (a mixed population containing both primitive progenitors and committed cells) (Fig. 1a,b), implying that miR-22 is upregulated during normal myelopoiesis. Similarly, we showed that miR-22 was also expressed at a significantly higher level in mouse normal bone marrow (BM) myeloid (Gr-1^+^/Mac-1^+^) cells, relative to lineage negative (Lin^−^) progenitor cells, long-term hematopoietic stem cells (LT-HSCs), short-term HSCs (ST-HSCs), and committed progenitors (CPs) (Supplementary Fig. 1e), further suggesting that miR-22 is upregulated in normal myelopoiesis.

### The anti-tumour effect of miR-22 in the pathogenesis of AML

Through bone marrow transplantation (BMT) assays, we showed that forced expression of miR-22 (but not miR-22 mutant) dramatically blocked *MLL-AF9* (MA9)-mediated leukemogenesis in primary BMT recipient mice, with a more potent inhibitory effect than miR-150 (Fig. 1e; Supplementary Fig. 2a). All MA9+miR-22 mice exhibited normal morphologies in peripheral blood (PB), BM, spleen and liver tissues (Fig. 1f), with a substantially reduced c-Kit^+^ blast cell population in BM (Supplementary Fig. 2b). Forced expression of miR-22 also almost completely inhibited leukemogenesis induced by *MLL-AF10* (Fig. 1g; Supplementary Fig. 2a). Conversely, miR-22 knockout significantly promoted *AML1-ETO9a* (AE9a)-induced AML (Fig. 1h). Thus, the repression of miR-22 is critical for the development of primary AML. Notably, forced expression of miR-22 in *MLL-AF9* and *MLL-AF10* leukaemia mouse models caused only a 2–3-fold increase in miR-22 expression level (Supplementary Fig. 2a), in a degree comparable to the difference in miR-22 expression levels between human AML samples and normal controls (Fig. 1a), suggesting that a 2–3-fold change in miR-22 expression level appears to be able to exert significant physiological or pathological effects.

**Figure 2:**
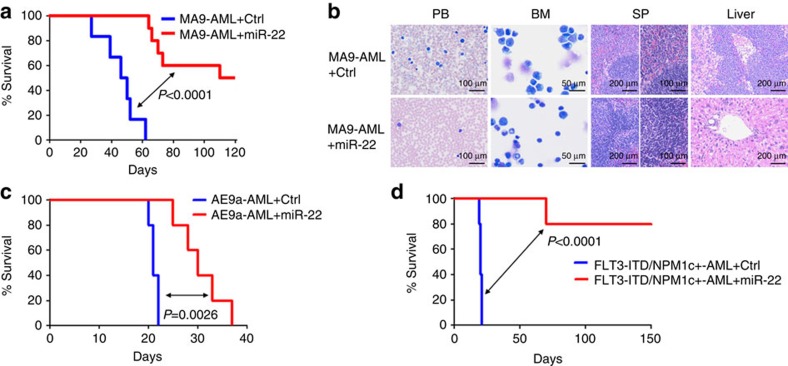
**Effect of miR-22 on the maintenance of AML** ***in vivo***. (**a,b**) Effect of miR-22 on the maintenance of *MLL-AF9*-induced AML in secondary BMT recipient mice. The secondary BMT recipients were transplanted with BM blast cells from the primary *MLL-AF9* AML mice retrovirally transduced with MSCV-PIG+MSCVneo (MA9-AML+Ctrl; *n*=7) or MSCV-PIG+MSCVneo-miR-22 (MA9-AML+miR-22; *n*=10). Kaplan–Meier curves (**a**) and Wright–Giemsa or H&E-stained PB, BM, spleen and liver (**b**) of the secondary leukaemic mice are shown. (**c,d**) Effect of miR-22 on the maintenance/progression of *AML1-ETO9a* (*AE9a*)-induced AML (**c**) or *FLT3*-ITD/*NPM1c*^+^-induced AML (**d**) in secondary BMT recipient mice (*n*=5 for each group). Kaplan–Meier curves and *P* values (log-rank test) are shown.

To examine whether the maintenance of AML is also dependent on the repression of miR-22, we performed secondary BMT assays. Forced expression of miR-22 remarkably inhibited progression of *MLL-AF9*-, *AE9a*- or *FLT3*-ITD/*NPM1c*^+^-induced AML in secondary recipient mice (Fig. 2a–d), resulting in largely normal morphologies in PB, BM, spleen and liver tissues (Fig. 2b; Supplementary Fig. 2c). Collectively, our findings demonstrate that miR-22 is a pivotal anti-tumour gatekeeper in both development and maintenance of various AML.

### Identification of critical target genes of miR-22 in AML

To identify potential targets of miR-22 in AML, we performed a series of data analysis. Analysis of In-house_81S (ref. 21) and TCGA_177S (ref. 22) data sets revealed a total of 999 genes exhibiting significant inverse correlations with miR-22 in expression. Of them, 137 genes, including 21 potential targets of miR-22 as predicted by TargetScan^18^ (Supplementary Table 1), were significantly upregulated in both human and mouse AML compared with normal controls as detected in two additional in-house data sets^14^,^23^. Among the 21 potential targets, *CRTC1*, *ETV6* and *FLT3* are known oncogenes^24^,^25^,^26^,^27^,^28^,^29^. We then focused on these three genes, along with *MYCBP* that encodes the MYC-binding protein and is an experimentally validated target of miR-22 (ref. 30) although due to a technical issue it was not shown in the 21-gene list (Supplementary Table 1), for further studies.

**Figure 3:**
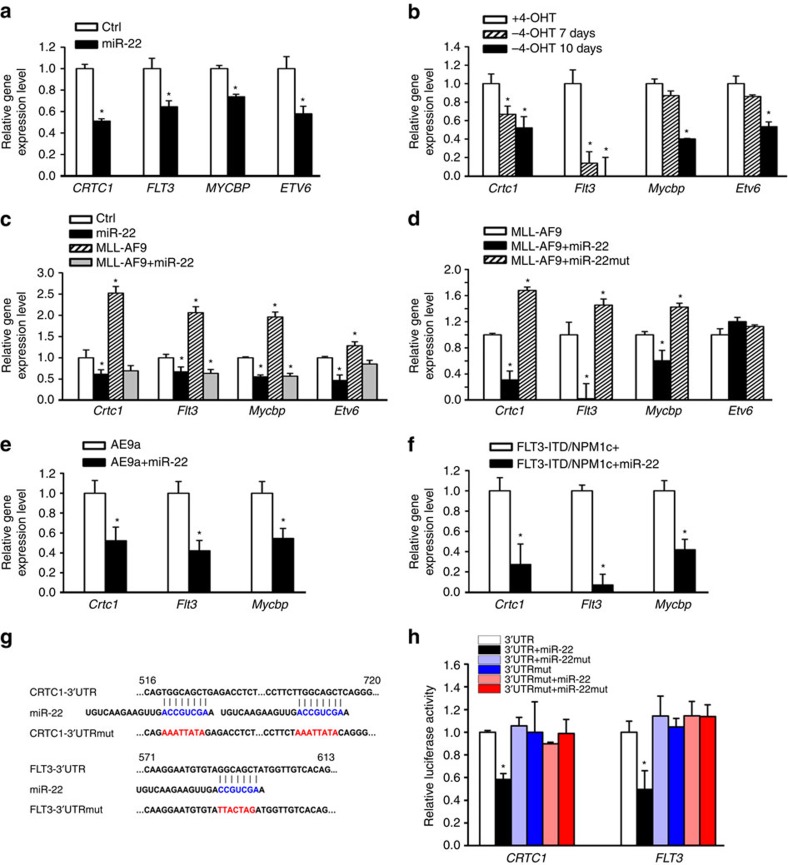
miR-22 targets multiple oncogenes. (**a**) Downregulation of *CRTC1*, *FLT3*, *MYCBP* and *ETV6* by forced expression of miR-22 in MONOMAC-6 cells. Expression of these genes was detected 48 h post transfection of MSCV-PIG (Ctrl) or MSCV-PIG-miR-22 (miR-22). (**b**) *Crtc1*, *Flt3*, *Mycbp* and *Etv6* levels in *MLL-ENL*-ERtm cells after withdrawal of 4-OHT for 0, 7 or 10 days. (**c**) Expression levels of *Crtc1*, *Flt3*, *Mycbp* and *Etv6* in mouse BM progenitor cells retrovirally transduced with MSCV-PIG+MSCV-neo (Ctrl), MSCV-PIG-miR-22+MSCV-neo (miR-22), MSCV-PIG+MSCV-neo-*MLL-AF9* (MLL-AF9) or MSCV-PIG-miR-22+MSCV-neo-*MLL-AF9* (MLL-AF9+miR-22). (**d**) Expression levels of *Crtc1*, *Flt3*, *Mycbp* and *Etv6* in BM blast cells of leukaemic mice transplanted with *MLL-AF9*, *MLL-AF9*+miR-22 or *MLL-AF9*+miR-22mut primary leukaemic cells. (**e,f**) Expression levels of *Crtc1*, *Flt3* and *Mycbp* in BM blast cells of leukaemic mice transplanted with MSCV-PIG or MSCV-PIG-miR-22-retrovirally transduced *AE9a* (**e**) or *FLT3*-ITD/*NPM1c*^+^ (**f**) primary leukaemic cells. (**g**) Putative miR-22 target sites and mutants in the 3′UTRs of *CRTC1* (upper panel) and *FLT3* (lower panel). (**h**) Effects of miR-22 on luciferase activity of the reporter gene bearing wild type or mutant 3′UTRs of *CRTC1* or *FLT3* in HEK293T cells. The mean±s.d. values from three replicates are shown. *P<0.05, *t*-test.

As expected, all four genes were significantly downregulated in expression by ectopic expression of miR-22 in human MONOMAC-6/t(9;11) cells (Fig. 3a). The coincidence of downregulation of those genes and upregulation of miR-22 was also observed in mouse *MLL-ENL*-ERtm cells, a leukaemic cell line with an inducible *MLL-ENL* derivative^31^, when MLL-ENL was depleted by 4-hydroxy-tamoxifen (4-OHT) withdrawal (Fig. 3b; Supplementary Fig. 3a). While *MLL-AF9* remarkably promoted expression of those four genes in mouse BM progenitor cells, co-expressed miR-22 reversed the upregulation (Fig. 3c). In leukaemia BM blast cells of mice with *MLL-AF9*-induced AML, the expression of *Crtc1*, *Flt3* and *Mycbp*, but not *Etv6*, was significantly downregulated by co-expressed miR-22 (but not by miR-22 mutant) (Fig. 3d). Because miR-22-mediated downregulation of *Etv6* could be observed only in the *in vitro* models (Fig. 3a–c), but not in the *in vivo* model (Fig. 3d), which was probably due to the difference between *in vitro* and *in vivo* microenvironments, we decided to focus on the three target genes (that is, *Crtc1, Flt3* and *Mycbp*) that showed consistent patterns between *in vitro* and *in vivo* for further studies. The repression of *Crtc1, Flt3* and *Mycbp* was also found in leukaemia BM cells of mice with *AE9a* or *FLT3*-ITD/*NPM1c*^+^-induced AML (Fig. 3e,f). As *Mycbp* is already a known target of miR-22 (ref. 30), here we further confirmed that *FLT3* and *CRTC1* are also direct targets of miR-22 (Fig. 3g,h). The downregulation of CRTC1, FLT3 and MYCBP by miR-22 at the protein level was confirmed in both human and mouse leukaemic cells (Supplementary Fig. 3b,c). Overexpression of miR-22 had no significant influence on the level of leukaemia fusion genes (Supplementary Fig. 3d).

**Figure 4:**
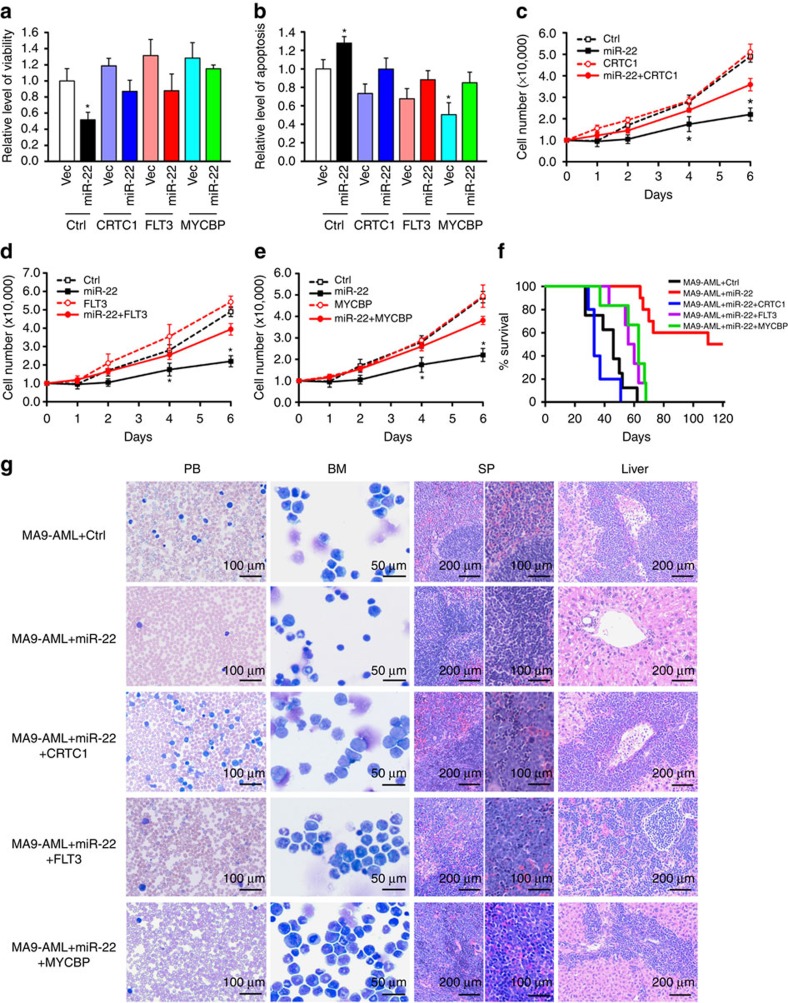
Multiple onocgenes are functionally important targets of miR-22 in AML. (**a,b**) Relative viability (**a**) and apoptosis (**b**) levels of MONOMAC-6 cells transfected with MSCV-PIG-*CRTC1, -FLT3* or -*MYCBP* alone, or together with MSCVneo-miR-22. Values were detected 48 h post transfection. (**c–e**) Rescue effects of *CRTC1* (**c**), *FLT3* (**d**) and *MYCBP* (**e**) on the inhibition of MONOMAC-6 growth mediated by miR-22. Cell counts at the indicated time points are shown. Mean±s.d. values are shown. *P<0.05, *t*-test. (**f**) *In vivo* rescue effects of *CRTC1*, *FLT3* and *MYCBP* on the inhibition of *MLL-AF9*-induced leukemogenesis mediated by miR-22. The secondary recipients were transplanted with BM blast cells of the primary *MLL-AF9* leukaemic mice retrovirally transduced with MSCVneo+MSCV-PIG (MA9-AML+Ctrl; *n*=7), MSCVneo-miR-22+MSCV-PIG (MA9-AML+miR-22; *n*=10), MSCVneo-miR-22+MSCV-PIG-*CRTC1* (MA9-AML+miR-22+CRTC1; *n*=5), MSCVneo-miR-22+MSCV-PIG-*FLT3* (MA9-AML+miR-22+FLT3; *n*=6) or MSCVneo-miR-22+MSCV-PIG-*MYCBP* (MA9-AML+miR-22+MYCBP; *n*=6). Kaplan–Meier curves for all the five groups of transplanted mice are shown. MA9-AML+Ctrl versus MA9-AML+miR-22, *P*<0.001 (log-rank test); MA9-AML+Ctrl versus any other groups, *P*>0.05 (log-rank test). (**g**) Wright–Giemsa stained PB and BM, and H&E stained spleen and liver of the secondary leukaemic mice.

Co-expression of the coding region (CDS) of each of the three target genes (that is, *CRTC1, FLT3* and *MYCBP*) largely reversed the effects of miR-22 on cell viability, apoptosis and proliferation (Fig. 4a–e). More importantly, *in vivo* BMT assays showed that co-expressing *CRTC1*, *FLT3* or *MYCBP* largely rescued the inhibitory effect of miR-22 on leukemogenesis (Fig. 4f,g; Supplementary Fig. 3e). Our data thus suggest that *CRTC1, FLT3* and *MYCBP* are functionally important targets of miR-22 in AML.

### miR-22 represses both CREB and MYC signalling pathways

CRTC1, a CREB-regulated transcription coactivator, facilitates CREB in regulating transcription of its targets, in both normal and malignant hematopoiesis^24^,^25^,^26^. *CDK6, HOXA7* and *RGS2* are known direct targets of CREB that are either positively (*CDK6* and *HOXA7*) or negatively (*RGS2*) regulated by CREB^32^,^33^,^34^,^35^. In both In-house_81S (ref. 21) and TCGA_177S (ref. 22) data sets, *CDK6* and *HOXA7* inversely, while *RGS2* positively, correlated with miR-22 in expression (Supplementary Table 2; Supplementary Fig. 3f). In leukaemic BM blast cells from primary and secondary BMT recipients, overexpression of miR-22 (but not miR-22 mutant) significantly downregulated expression of *Cdk6* and *Hoxa7*, while upregulating *Rgs2*, which could be reversed by co-expressing *CRTC1* (Supplementary Fig. 3g,h). These results suggest that miR-22 represses the CREB signalling pathway in AML by targeting *CRTC1*.

MYCBP, a MYC-binding protein, is essential for MYC-mediated gene regulation^30^. *FLT3* is an upstream regulator of *MYC*^17^. In leukaemic BM cells, forced expression of miR-22, but not miR-22 mutant, significantly repressed expression of MYC downstream oncogenic targets *Bmi1, Fasn* and *Hmga1* (refs 36, 37, 38); the repression could be reversed by co-expressing *MYCBP* or *FLT3* (Supplementary Fig. 3i,j). Those three genes all showed significant inverse correlations with miR-22 in expression in human AML (Supplementary Table 2; Supplementary Fig. 3f). The miR-22-induced repression of Bmi1, Cdk6 and Hmga1 at the protein level was also observed (Supplementary Fig. 3c).

### DNA copy-number loss of miR-22 gene locus in AML

DNA copy-number loss of tumour-suppressor gene(s) is a hallmark of many cancers including AML^39^. Deletions of human chromosome 17 band p13.3, where miR-22 is located, have been frequently reported in various types of leukaemia, lymphoma and solid tumours^40^,^41^,^42^,^43^. Here we found that 18% (9/50) of the AML samples showed deletions (mostly hemizygous) of the miR-22 gene locus (Supplementary Fig. 4a,b). Similarly, in analysis of three publically available AML data sets, we found that 7–9% of the AML cases carried loss of one or even two alleles of the miR-22 locus (Supplementary Fig. 4c). Therefore, DNA copy-number loss in miR-22 gene locus does exist in AML cases.

### Expression of miR-22 is epigenetically repressed in AML

**Figure 5:**
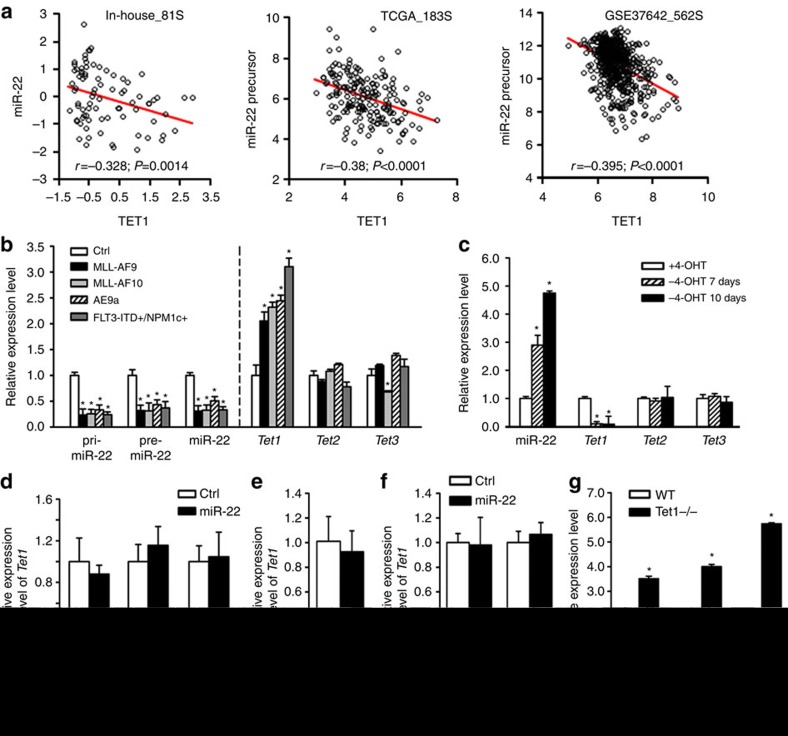
**Transcriptional correlation between miR-22 and** ***TET1***. (**a**) Correlation between the expression levels of miR-22 and *TET1* in three independent AML patient databases. All expression data were log(2) transformed; the data in In-house_81S were also mean-centred. The correlation coefficient (*r*) and *P* values were detected by ‘Pearson Correlation’, and the correlation regression lines were drawn with the ‘linear regression’ algorithm. (**b**) Expression of pri-, pre- and mature miR-22, and *Tet1/2/3* in colony-forming cells of wild-type mouse BM progenitors retrovirally transduced with MSCVneo (Ctrl), MSCVneo-*MLL-AF9* (MLL-AF9), MSCVneo-*MLL-AF10* (MLL-AF10) or MSCVneo-*AE9a* (AE9a), or of *FLT3*-ITD/*NPM1c*^*+*^ mouse BM progenitors transduced with MSCVneo (FLT3-ITD+/NPM1c+). (**c**) Expression of miR-22 and *Tet1/2/3* in *MLL-ENL*-ERtm cells. Expression levels were detected at the indicated time points post 4-OHT withdrawal. (**d**) Effect of miR-22 overexpression on *Tet1* expression in colony-forming cells with *MLL-AF9*, *AE9a* or *FLT3*-ITD/*NPM1c*^+^. (**e**) Expression of *Tet1* in BM progenitor cells of 6-weeks old miR-22^−/−^ or wild-type mice. (**f**) Effect of miR-22 overexpression on *TET1* expression in THP-1 and KOCL-48 AML cells 48 h post transfection. (**g**) Expression of pri-, pre- and mature miR-22 in BM progenitor cells of 6-weeks old *Tet1*^−/−^ or wild-type mice. Mean±s.d. values are shown. *P<0.05, *t*-test.

It was reported that *TET2* is repressed by miR-22 as its direct target in breast cancer and MDS^15^,^16^. Here we analysed the expression patterns of *TET1/2/3* and miR-22 in three independent AML patient data sets^21^,^22^,^44^ (Supplementary Table 3). To our surprise, we found that *TET2* (and likely also *TET3*) exhibited a positive correlation, whereas only *TET1* exhibited a negative correlation, with miR-22 in expression in AML (Supplementary Table 3; Fig. 5a). The primary, precursor and mature miR-22 levels were all significantly downregulated by *MLL-AF9*, *MLL-AF10, AE9a* and *FLT3*-ITD/*NPM1c*^*+*^ in colony-forming cells, while *Tet1* (but not *Tet2* or *Tet3*) was upregulated (Fig. 5b). Conversely, in *MLL-ENL*-ERtm cells^31^, *Tet1*, but not *Tet2* or *Tet3*, was downregulated when miR-22 was upregulated after withdrawal of 4-OHT (Fig. 5c). Thus, *Tet1*, instead of *Tet2*, exhibited an inverse correlation with miR-22 in expression in both human and mouse leukaemic cells. *Tet1* also exhibits an inverse correlation with miR-22 in expression during mouse normal myeloid differentiation (Supplementary Fig. 5a). Furthermore, as miR-22 and *TET1* were expressed at a significantly higher and lower level, respectively, in human normal CD33^+^ myeloid progenitor cells than in CD34^+^ HSPCs or MNCs (see Fig. 1a and ref. 14), the inverse expressional correlation between miR-22 and *TET1* likely also existed in human normal hematopoietic cells.

However, forced expression of miR-22 caused no noticeable changes in *Tet1* expression in *MLL-AF9*, *AE9a* or *FLT3*-ITD/*NPM1c*^+^ colony-forming cells (Fig. 5d). Similarly, neither miR-22 knockout nor overexpression resulted in any significant changes of *Tet1/TET1* expression (Fig. 5e,f). In contrast, *Tet1* knockout remarkably increased the levels of pri-, pre- and mature miR-22 (Fig. 5g). Thus, our data suggest that miR-22 is a downstream target of and negatively regulated by Tet1, and that there is no negative feedback of miR-22 on *Tet1* expression.

**Figure 6:**
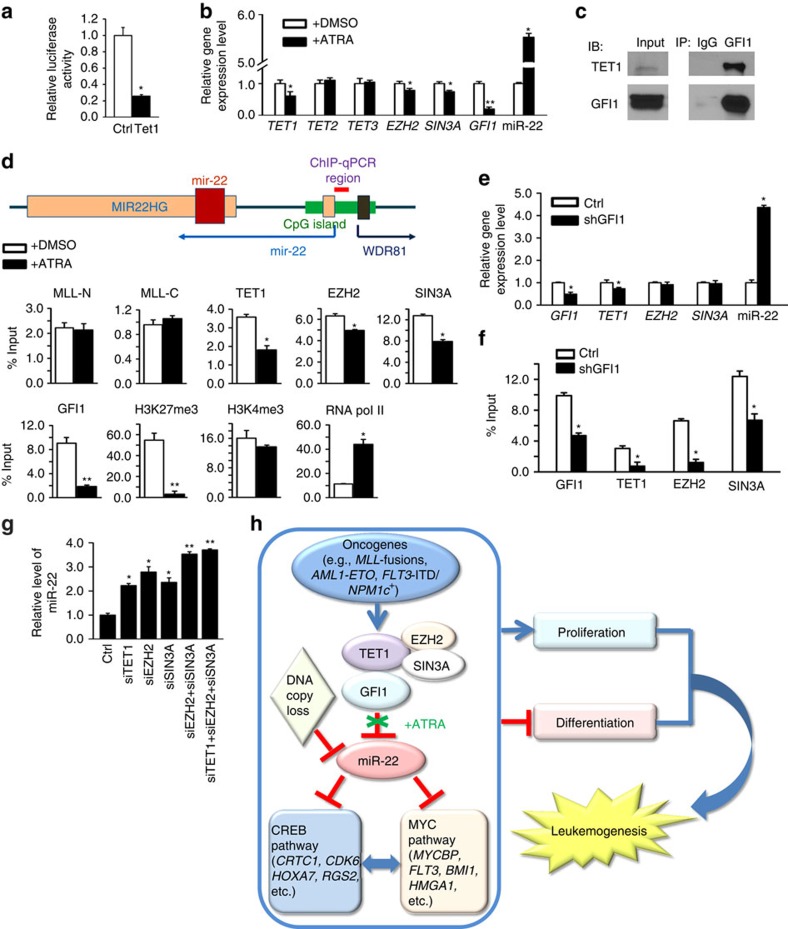
TET1 mediates epigenetic repression of miR-22 transcription. (**a**) Tet1 targets miR-22 promoter region (−1,100/+55 bp), as detected by luciferase reporter assay 48 h post transfection in HEK293T cells. (**b**) Expression of *TET1/2/3*, *EZH2*, *SIN3A*, *GFI1* and miR-22 in THP-1 cells 72 h post treatment with 1 μM ATRA or DMSO control. (**c**) Co-immunoprecipitation assay showing the binding of endogenous GFI1 and TET1 in THP1 cells. (**d**) ChIP-qPCR analyses of the promoter region of miR-22 in THP-1 cells 72 h post treatment with 1 μM ATRA or DMSO. Upper panel: PCR site on the CpG-enriched region of miR-22 gene locus. Note: miR-22 is coded within the second exon of a long non-coding RNA (MIR22HG), which represents the primary transcript of miR-22. Lower panels: enrichment of MLL-N terminal (for both wild-type MLL and MLL-fusion proteins), MLL-C terminal (for wild-type MLL), TET1, EZH2, SIN3A, GFI1, H3K27me3, H3K4me3 or RNA pol II at miR-22 promoter region. (**e**) Expression levels of TET1, EZH2, SIN3A and miR-22 in GFI1 knockdown cells. (**f**) ChIP-qPCR analyses of the promoter region of miR-22 in THP-1 cells transduced with GFI1 shRNA or control shRNA. Enrichment of GFI1, TET1, EZH2 and SIN3A are shown. (**g**) Effects of knockdown of *TET1*, *EZH2* and/or *SIN3A* on miR-22 expression. The expression level of miR-22 was detected in THP-1 cells 72 h post transfection with siRNAs targeting *TET1*, *EZH2* and/or *SIN3A*. Mean±s.d. values are shown. *P<0.05; **P<0.01 (*t*-test). (**h**) Schematic model of the regulatory pathway involving miR-22 in AML and ATRA treatment.

Tet1 has been shown to cooperate with Polycomb repressive complex 2 (PRC2) components and cofactors, such as Ezh2 and Sin3a, to repress transcription of their co-target genes in mouse embryonic stem cells^8^,^9^. Our luciferase reporter assay showed that forced expression of Tet1 significantly repressed the transcriptional activity controlled by the miR-22 promoter^45^, suggesting that miR-22 is a direct repressed target of Tet1 (Fig. 6a). In an all-*trans* retinoic acid (ATRA)-induced THP-1/t(9;11) monocytic differentiation model^46^, we showed that on treatment with ATRA, *TET1* (but not *TET2* or *TET3*), *EZH2* and *SIN3A* were significantly downregulated, accompanied by the upregulation of miR-22 (Fig. 6b). *WDR81* is the gene that is located closely (within 500 bp) but oppositely to the miR-22 gene loci (Fig. 6d). We also tested the potential influence of ATRA on the expression level of *WDR81* in the same model. ATRA treatment showed no significant effects on *WDR81* level (Supplementary Fig. 5b), suggesting that TET1 specifically inhibits the transcription of miR-22, but not its neighbouring gene with the opposite orientation.

While miR-22 expression level had a more than fivefold increase on ATRA treatment, the degrees of decrease in expression levels of *TET1*, *EZH2* and *SIN3A* are relatively mild (though statistically significant) (Fig. 6b). To identify additional transcription factor(s) that is (are) more responsive to ATRA treatment and can facilitate TET1 binding to miR-22 promoter region, we searched for transcription factors that have evolutionarily conserved binding sites within the CpG island of miR-22 locus. Among a set of such transcription factors (including GFI1, STAT, PAX4, HMX1 and SRF), only *GFI1* exhibited a significant inverse correlation with miR-22 in expression in all large-scale AML cohorts (Supplementary Table 3). Interestingly, it was reported previously that ATRA treatment could significantly diminish the binding of GFI1 to the loci of many of its target genes, for example, *IL-6R, JAK3*, *E2F6* and so on^47^. Thus, we chose GFI1 for further studies.

Notably, we found that ATRA treatment substantially reduced the transcription level of *GFI1* in AML cells and its decrease degree was greater than that of *TET1*, *EZH2* or *SIN3A* (Fig. 6b). We further showed that GFI1 is a binding partner of TET1 in both THP1 and HEK-293T cells (Fig. 6c; Supplementary Fig. 5c). ATRA treatment remarkably reduced the binding of GFI1, TET1, EZH2 and SIN3A, but not that of MLL protein, to the miR-22 promoter region (Fig. 6d). H3K27me3 modifications and RNA polymerase II (RNA pol II) occupancy were significantly decreased and increased, respectively, while H3K4Me3 modifications showed no significant change (Fig. 6d). Noticeably, the enrichment of GFI1 to this region was diminished by ATRA to a greater degree than that of TET1, EZH2 or SIN3A (Fig. 6d), suggesting that GFI1 might be the primary effector of ATRA treatment in regulating miR-22 expression. Consistently, knockdown of *GFI1* resulted in a dramatic increase in miR-22 expression (>4 fold; Fig. 6e), associated with a significant decrease in the binding of TET1, EZH2, SIN3A and GFI1 itself to the miR-22 promoter region (Fig. 6f). Knockdown of expression of *TET1*, *EZH2* or *SIN3A* resulted in a 2–3-fold increase in miR-22 expression (Fig. 6g), with no effects on *GFI1* expression (Supplementary Fig. 5d); only their combinational knockdown could cause a similar level of increase in miR-22 expression (Fig. 6g; Supplementary Fig. 5e) to that induced by *GFI1* knockdown (Fig. 6e). As expected, the expression level of *WDR81* was not changed on knockdown of *GFI1*, *TET1, EZH2* or *SIN3A* (Supplementary Fig. 5f,g). The above data suggest that GFI1, TET1, EZH2 and SIN3A are all involved in transcriptional repression of miR-22 expression; and when treated with ATRA, GFI1 likely functions as the primary effector that facilitates the binding of TET1/EZH2/SIN3A complex to the miR-22 promoter region.

As TET1 is a methylcytosine dioxygenase^8^,^9^,^10^, we conducted bisulfite sequencing analysis to investigate whether TET1 affects the methylation status of the miR-22 promoter. The analysis showed that the miR-22 promoter was hypomethylated in AML cells, no matter with ATRA treatment or not (Supplementary Fig. 5h). The hypomethylated status of the miR-22 promoter region in various AML was confirmed by analysing the TCGA_194S data set with DNA methylation information (Supplementary Fig. 5i). The methylation status of the miR-22 promoter showed no significant correlation with miR-22 expression level in AML (Supplementary Fig. 5j). These data suggest that the hypomethylation status of miR-22 promotor region does not lead to a high-level expression of miR-22 in AML, and TET1-mediated repression of miR-22 transcription is unlikely related to its methylcytosine dioxygenase activity.

### The miR-22-associated regulatory circuit in AML

The above data suggest that repression of miR-22 in AML is attributed to both DNA copy-number loss and especially TET1-mediated transcriptional suppression. Interestingly, among the nine AML samples with DNA copy-number loss of miR-22 locus (Supplementary Fig. 4a,b), the AML samples with both copy-number loss and TET1 overexpression generally exhibited a more significant repression of miR-22 expression than those with copy-number loss alone (Supplementary Fig. 5k). Thus, those two mechanisms are not mutually exclusive and can have synergistic effect on reducing miR-22 expression.

Collectively, our studies revealed a previously unappreciated genetic/epigenetic regulatory circuit in AML (Fig. 6h). In this circuit, oncogenic fusion genes or gene mutants (for example, *MLL*-fusions, *AE9a* and *FLT3*-ITD/*NPM1c*^+^) function as the ‘drivers’. They promote the expression of *TET1*, which in turn, through recruiting polycomb cofactors such as EZH2 and SIN3A, represses the transcription of miR-22 by increasing H3K27me3 and decreasing RNA Pol II binding at the miR-22 promoter. When AML cells are treated with ATRA, ATRA substantially diminishes the enrichment of GFI1, a binding partner of TET1, at the miR-22 promoter, and thereby inhibits the recruitment of the TET1/EZH2/SIN3A complex to this region. In addition, miR-22 can also be compromised in its function by genetic mechanism(s) such as DNA copy-number loss in a portion (7–18%) of the AML cases. The inactivation of miR-22 results in the de-repression of its critical oncogenic targets such as *CRCT1*, *MYCBP* and *FLT3*, and thereby the activation of both CREB and MYC signalling pathways, leading to cell transformation and leukemogenesis.

### Restoration of miR-22 expression and function to treat AML

**Figure 7:**
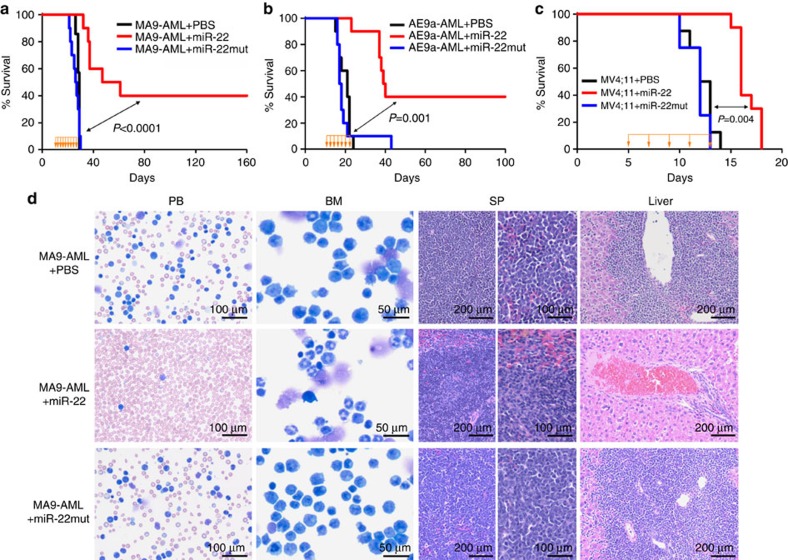
Therapeutic effect of miR-22-nanoparticles in treating AML. (**a,b**) Primary leukaemia BM cells bearing *MLL-AF9* (**a**) or *AE9a* (**b**) were transplanted into sublethally irradiated secondary recipient mice. After the onset of secondary AML (usually 10 days post transplantation), the recipient mice were treated with PBS control, or 0.5 mg kg^−1^ miR-22 or miR-22 mutant RNA oligos formulated with G7 PAMAM dendrimer nanoparticles, i.v., every other day, until the PBS-treated control group all died of leukaemia. (**c**) NSGS mice^49^ were transplanted with MV4;11/t(4;11) AML cells. Five days post transplantation, these mice started to be treated with PBS control, miR-22 or miR-22 mutant nanoparticles at the same dose as described above. Kaplan–Meier curves are shown; the drug administration period and frequency were indicated with yellow arrows. The *P* values were detected by log-rank test. (**d**) Wright–Giemsa stained PB and BM, and H&E stained spleen and liver of the *MLL-AF9*-secondary leukaemic mice treated with PBS control, miR-22 or miR-22 mutant nanoparticles.

To investigate the therapeutic potential of restoration of miR-22 expression/function in treating AML, we employed amine-terminated, generation 7 (G7) poly(amidoamine) (PAMAM) dendrimers (Supplementary Fig. 6a,b), an effective non-viral gene delivery vector with minimal side effects^48^. Nanoparticles carrying miR-22 oligos significantly delayed AML progression in both *MLL-AF9* and *AE9a*-induced secondary leukaemic recipients (Fig. 7a,b). Notably, at least 40% of the treated mice seemed to be completely cured by the miR-22 nanoparticles as the pathological morphologies in PB, BM, spleen and liver tissues all became normal (Fig. 7d; Supplementary Fig. 6c). In contrast, the miR-22 mutant nanoparticles exhibited no significant therapeutic effect (Fig. 7a,b,d; Supplementary Fig. 6c). As expected, miR-22 oligos, but not miR-22 mutant oligos, significantly inhibited expression of its critical targets (that is, *Crct1*, *Flt3* and *Mycbp*) in BM cells of the treated mice (Supplementary Fig. 6d). The miR-22-nanoparticles showed no noticeable effects on blood cell lineages (Supplementary Table 4).

We then tested the miR-22 nanoparticles in a xeno-transplantation model^49^. Similarly, the nanoparticles carrying miR-22 oligos, but not miR-22 mutant, significantly delayed AML progression induced by human MV4;11/t(4;11) cells (Fig. 7c). The miR-22-nanoparticle administration also resulted in less aggressive leukaemic pathological phenotypes in the recipient mice (Supplementary Fig. 6e). Thus, our studies demonstrated the therapeutic potential of using miR-22-based nanoparticles to treat AML.

## Discussion

It remains poorly understood how TET proteins mediate gene regulation in cancer. Here we show that in *de novo* AML, it is TET1, but not TET2 (a reported direct target of miR-22 in MDS and breast cancer^15^,^16^), that inversely correlates with miR-22 in expression and negatively regulates miR-22 at the transcriptional level. Likely together with GFI1, TET1 recruits polycomb cofactors (for example, EZH2/SIN3A) to the miR-22 promoter, leading to a significant increase in H3K27me3 occupancy and decrease in RNA pol II occupancy at that region, and thereby resulting in miR-22 repression in AML cells; such a repression can be abrogated by ATRA treatment. Thus, our study uncovers a novel epigenetic regulation mechanism in leukaemia involving the cooperation between TET1/GFI1 and polycomb factors.

Besides GFI1, it was reported that LSD1 is also a binding partner of TET1 (ref. 50). Interestingly, LSD1 is known as a common binding partner shared by TET1 and GFI1, and mediates the effect of GFI1 on hematopoietic differentiation^51^,^52^. Thus, it is possible that LSD1 might also participate in the transcriptional repression of miR-22 as a component of the GFI1/TET1 repression complex.

We previously reported that TET1 cooperates with MLL fusions in positively regulating their oncogenic co-targets in *MLL*-rearranged AML^14^. Here we show that TET1 can also function as a transcriptional repressor (of a miRNA) in cancer. The requirement of TET1-mediated regulation on expression of its positive (for example, *HOXA/MEIS1/PBX3*)^14^ or negative (for example, miR-22) downstream effectors in leukemogenesis likely explains the rareness of *TET1* mutations in AML^53^, and highlights its potent oncogenic role in leukaemia.

The aberrant activation of both CREB and MYC signalling pathways has been shown in AML^24^,^25^,^26^,^54^,^55^, but the underlying molecular mechanisms remain elusive. Our data suggest that the activation of these two signalling pathways in AML can be attributed, at least in part, to the repression of miR-22, which in turn, results in the de-repression of *CRTC1* (CREB pathway), *FLT3* and *MYCBP* (MYC pathway), and leads to the upregulation of oncogenic downstream targets (for example, *CDK6*, *HOXA7*, *BMI1*, *FASN* and *HMGA1*) and downregulation of tumour-suppressor downstream targets (for example, *RGS2*).

In summary, we uncover a TET1/GFI1/EZH2/SIN3A⊣miR-22⊣CREB-MYC signalling circuit in *de novo* AML, in which miR-22 functions as a pivotal anti-tumour gate-keeper, distinct from its oncogenic role reported in MDS or MDS-derived AML^16^. Thus, our study together with the study of Song *et al*.^16^ highlight the complexity and functional importance of miR-22-associated gene regulation and signalling pathways in hematopoietic malignancies, and may provide novel insights into the genetic/epigenetic differences between *de novo* AML and MDS.

Our findings also highlight the possibility of using miR-22-based therapy to treat AML patients. Our proof-of-concept studies demonstrate that the nanoparticles carrying miR-22 oligos significantly inhibit AML progression and prolong survival of leukaemic mice in both BMT and xeno-transplantation models. Notably, miRNA-based nanoparticles have already entered clinical trials^56^. It would be important, in the future, to further test the combination of miR-22-carrying nanoparticles (or small-molecule compounds that can induce endogenous expression of miR-22) with standard chemotherapy agents (cytosine arabinoside and anthracycline), or with the emerging small molecule inhibitors against MYC and/or CREB pathway effectors, to achieve optimal anti-leukaemia effect with minimal side effects. Overall, our results suggest that restoration of miR-22 expression/function (for example, using miR-22-carrying nanoparticles or small-molecule compounds) holds great therapeutic potential to treat AML, especially those resistant to current therapies.

## Methods

### AML and MDS samples and cell lines

The AML and MDS patient samples were obtained at the time of diagnosis with informed consent at the University of Chicago Hospital (UCH), and were approved by the University of Chicago Hospital Institutional Review Board (UCHIRB). All patients were treated according to the protocols of the corresponding institutes/hospitals. THP-1, KOCL48, MV4;11, MEF and HEK-293T cells were purchased from ATCC (Manassas, VA) and maintained in the lab. The MLL-ENL-ER cell line was a gift from Dr Robert Slany^31^. All the cell lines were tested for mycoplasma contamination yearly using a PCR Mycoplasma Test Kit (PromoKine).

### Preparation of AML and MDS samples

The primary AML and MDS samples were stored in liquid nitrogen until used. Blasts and mononuclear cells were purified by use of NycoPrep 1.077A (Axis-Shield, Oslo, Norway) according to the manufacturer’s manual.

### Human normal hematopoietic control cell samples

The MNC normal control samples were isolated from normal BM cells purchased from AllCells, LLC (Emeryville, CA) by use of NycoPrep 1.077A (Axis-Shield, Oslo, Norway) according to the manufacturer’s manual.

### Mouse normal BM cell population sorting

As described previously^23^, wild-type C57BL6/J mice were used for the sorting. All laboratory mice were maintained in the animal facility at the University of Chicago and the University of Cincinnati. All experiments on mice in our research protocol were approved by Institutional Animal Care and Use Committee (IACUC) of the University of Chicago and the University of Cincinnati.

The long-term hematopoietic stem cells (LT-HSCs; Lin^−^Sca1^+^c-Kit^+^Flk2^-^, LSKF^−^), short-term HSCs (ST-HSCs; Lin^−^Sca1^+^c-Kit^+^Flk2^+^, LSKF^+^), and the committed progenitors (CP, Lin^−^Sca1^−^c-Kit^+^) were enriched by lineage^+^ cell depletion (EasySep Mouse Hematopoietic Progenitor Cell Enrichment Kit; StemCell Technologies, Vancouver, BC), and purified by FACSAria flow cytometer (BD Biosciences, San Jose, CA) sorting after 20 μl per test fluorescein isothiocyanate-conjugated lineage (FITC-Lin) cocktail (including FITC-CD3 (17A2), FITC-B220 (RA3-6B2), FITC-CD11b (M1/70), FITC-TER-119 (TER-119), FITC-Gr1 (RB6-8C5)), 5 μg ml^−1^ phycoerythrin (PE)-Sca1 (D7), 1.5 μg ml^−1^ APC-c-Kit (ACK2) and 20 μg ml^−1^ PE-Cy5.5-Flk2 (A2F10) staining. Then, Gr1^+^Mac1^+^ myeloid cells and B220^+^ B cells were sorted from BM cells after 2.5 μg ml^−1^ FITC-Gr1 (RB6-8C5), 2.5 μg ml^−1^ PE-Mac1 (M1/70) and 5 μg ml^−1^ APC-B220 (RA3-6B2) staining. All fluorescent antibodies used were purchased from eBioscience (San Diego, CA).

### RNA extraction and quantitative RT–PCR

Total RNA was extracted with the miRNeasy extraction kit (Qiagen, Valencia, CA) and was used as template to synthesize complementary DNA for quantitative reverse transcription PCR (qRT–PCR) analysis in a 7900HT real-time PCR system (Applied Biosystems, Foster City, CA). TaqMan qPCR assay was performed to validate the differential expression patterns of miR-22 using commercial kits from Applied Biosystems (Cat. no. 4427975). Sequences for the controls are: sno202: 5′- GCTGTACTGACTTGATGAAAGTACTTTTGAACCCTTTTCCATCTGATG -3′; RNU6B: 5′- CGCAAGGATGACACGCAAATTCGTGAAGCGTTCCATATTTTT -3′. qPCR with SYBR Green dye (Qiagen) was used to determine expression of mRNA genes. *snoRNA202*, *RNU48*, *Gapdh* or *GAPDH* were used as endogenous controls for qPCR of miRNA and mRNA, respectively. Each sample was run in triplicate. qPCR primers are available on request. For determining the miR-22 DNA locus copy number, TaqMan qPCR assay was used as described previously^57^.

### microRNA microarray and exon array assays

As described previously^17^,^23^, our miRNA expression profiling assay of 85 (including 10 t(8;21), 9 inv(16), 9 t(15;17), 10 *MLL*-rearranged, 11 (+8), 29 normal karyotype and 7 others) AML samples, and 15 human normal BM samples was performed by Exiqon (Woburn, MA) using the miRCURY LNA arrays (v10.0; covering 757 human miRNAs). The 15 normal BM controls included six CD34+ hemtopoietic stem/progenitor, five CD33+ myeloid progenitor and four MNC samples. In terms of patient samples, MNCs isolated from the BM or PB cells of the 85 AML patients were used. The expression values are log2 (Hy3/Hy5) ratios, which were obtained on the basis of the normalized data where replicated measurements on the same slide have been averaged. In addition, as described previously^14^,^17^,^23^, a total of 100 human AML (including 30 t(8;21), 27 inv(16), 31 t(15;17) and 12 *MLL*-rearranged), and 9 normal BM samples (including three each of CD34^+^ hematopoietic stem/progenitor, CD33^+^ myeloid and MNC samples) were analysed by use of Affymetrix GeneChip Human Exon 1.0 ST arrays (Affymetirx, Santa Clara, CA). The QC test and Affymetrix exon array assays were done in the core facility of National Human Genome Research Institute, NIH (Bethesda, MD). Robust multi-array average (RMA)^58^ was used for the data normalization with Partek Genomics Suite (Partek Inc., St Louis, MI). The complete microarray data set has been deposited in the GEO database under the accession codes GSE34184 and GSE30285.

Among the above 100 human AML samples, 81 samples (that is, the In-house_81S; including 29 t(8;21), 26 inv(16) and 26 t(15;17) AML) have been also included in the Exiqon microRNA array assay^21^. The microarray data set of those 81 AML samples has been deposited in GEO database under the accession code GSE27370.

### Affymetrix gene arrays of mouse samples

As described previously^17^, a total of 15 mouse BM samples including 6 primary (including three each of negative control and *MLL-AF9*) and 9 secondary (including three negative control and six *MLL-AF9*) obtained from the *in vivo* mouse BM reconstitution assays were analysed by use of Affymetrix GeneChip Mouse Gene 1.0 ST Array (Affymetirx). The RNA quality control, cDNA amplification, hybridization and image scan were conducted in the Functional Genomics Facility of the University of Chicago. RMA^58^ was used for the data normalization with Partek Genomics Suite (Partek Inc.). The microarray data set of those 15 mouse AML samples has been deposited at GEO database (GSE34185)

### Affymetrix microarray assay of GSE37642_562S set

The GSE37642_562S set (*n*=562) AML samples (including 30 t(8;21), 38 inv(16), 24 t(15;17), 38 *MLL*-rearranged, 6 del(5q), 16 del(7q), 15 inv3/t(3/3), 74 complex, 199 normal karyotype and 122 others) were analysed by use of Affymetrix Human Genome U133Plus2.0 GeneChips (*n*=140) or Affymetrix Human Genome U133A and B (U133A+B; *n*=422) GeneChips. RMA method^58^ was used for data normalization. The AML samples were collected by the German AMLCG study group. Part of the microarray data have been reported previously^44^. The GEO ID of the entire data set is GSE37642.

### TCGA data sets

The Cancer Genome Atlas (TCGA) AML database^22^ includes mRNA gene expression profiling data of 183 adult *de novo* AML cases (that is, TCGA_183S; including 7 t(8;21), 11 inv(16), 17 t(15;17), 9 *MLL*-rearranged, 3 t(9;22), 22 complex, 78 normal karyotype and 36 others), which were generated by use of Affymetrix Human Genome U133Plus2.0 GeneChips. Among the 183 AML cases, 177 (including 7 t(8;21), 11 inv(16), 16 t(15;17), 9 *MLL*-rearranged, 3 t(9;22), 22 complex, 75 normal karyotype and 34 others) also have microRNA expression profiles as detected by IlluminaGA_miRNASeq platform, and the mRNA/miRNA profile data of the 177 AML cases were collectively referred to as TCGA_177S. One hundred ninety-four adult *de novo* AML cases (including 7 t(8;21), 11 inv(16), 15 t(15;17), 9 *MLL*-rearranged, 3 t(9;22), 24 complex, 91 normal karyotype and 34 others) with DNA methylation data as detected by Infinium HumanMethylation450 BeadChip were referred to as TCGA_194S. The mRNA/miRNA expression data and methylation data were downloaded from https://tcga-data.nci.nih.gov/tcga/dataAccessMatrix.htm? mode=ApplyFilter& showMatrix=true&diseaseType=LAML& 12:28 PM 4/6/2016tumorNormal=TN&tumorNormal=T&tumorNormal=NT.

### Cell culture and transfection

These experiments were conducted as described previously^17^,^23^ with some modifications. THP-1, KOCL-48 and MV4;11 cells were grown in RPMI medium 1640 (Invitrogen, Carlsbad, CA) containing 10% FBS, 1% HEPES and 1% penicillin–streptomycin. MONOMAC-6 cells were maintained in RPMI 1640 supplemented with 10% FBS, 1% HEPES, 2 mM L-glutamine, 100 × Non-Essential Amino Acid, 1 mM sodium pyruvate, 9 μg ml^−1^ insulin and 1% penicillin–streptomycin. Plasmids or siRNAs were transfected into MONOMAC-6 cells with Cell Line Nucleofector Kit V following program T-037, and THP-1 and KOCL-48 cells following program U-001, using the Amaxa Nucleofector Technology (Amaxa Biosystems, Berlin, Germany). Experiments were performed 48 h after transfection.

For the ATRA-treatment study, THP-1 cells were seeded at a concentration of 0.4 × 10^6^ ml^−1^ and treated with ATRA (1 μmol l^−1^) or vehicle control (DMSO, 0.001%) for 72 h before cells were collected for RNA analysis or chromatin immunoprecipitation (ChIP) assays.

The *MLL-ENL*-ERtm cell line was kept in RPMI 1640 supplemented with interleukin 3 (IL-3), IL-6 and granulocyte-macrophage colony-stimulating factor (GM-CSF), 10 ng ml^−1^; stem cell factor (SCF) 100 ng ml^−1^; 10% FBS and 1% penicillin–streptomycin. 4-OHT (Sigma-Aldrich, St Louis, MO) was added at a 100-nM final concentration as a 1-mM stock solution in ethanol. Cells were collected for experiments at the indicated days after drug withdrawal.

The MEF and HEK-293T cells were kept in DMEM (Invitrogen) containing 10% FBS, 1% HEPES and 1% penicillin–streptomycin; HEK-293T cells were transfected with Qiagen Effectene Transcription Kit (Qiagen) following the manufacturer’s protocol.

All the cell lines were mycoplasma negative.

### Lentivirus production and infection

All the plasmid for packaging lentivirus, including pMD2.G, pMDLg/pRRE and pRSV-Rev, were purchased from Addgene (Cambridge, MA). First, 0.5 μg pMD2.G, 0.3 μg pMDLg/pRRE, 0.7 μg pRSV-Rev and 1.5 μg shRNA constructs, that is shGFI1 and control (purchased from GE Dharmacon, Pittsburgh, PA) were co-transfected into HEK-293T cells in 60 mm cell culture dish with Effectene Transfection Reagent (Qiagen). The lentivirus particles were harvested at 48 and 72 h after transfection and concentrated with PEG-it Virus Precipitation Solution (SBI). Finally, the lentivirus particles were directly added into leukaemic cells and these cells were washed with PBS 24–48 h after infection.

### Viability and proliferation assays

These experiments were conducted as described previously^17^,^23^ with some modifications. For apoptosis and viability assays, 48 h after transfection, cells were collected and seeded with requested concentration. Cell apoptosis and viability were assessed using ApoLive-Glo Multiplex Assay Kit (Promega, Madison, WI) following the corresponding manufacturer’s manuals. For cell proliferation assays, per million cells were electroporated with 1.5 μg plasmid. Twenty-four hours after transfection, cells were seeded in 96-well plates at the concentration of 10,000 cells per well. Cell numbers were counted at the indicating days.

### Plasmid construction

The home-prepared expression vector of miR-22, that is MSCV-PIG-miR-22, was amplified by PCR using primers: forward: 5′- GCCCTCGAGTCTAGACTCCAGTTC -3′ and reverse: 5′- GGGGAATTCCTACTCCTCAATCCAG -3′, and was subsequently cloned into the XhoI and EcoRI sites of the retrovirus vector MSCV-PIG (that is, MSCV-puro-IRES-GFP vector; bearing *GFP* gene), a kind gift from Drs Gregory Hannon, Scott Hammond and Lin He (Cold Spring Harbor Laboratory, Cold Spring Harbor, NY). The other miR-22 construct, the MSCV-PIG-miR-22_2 expression vector, was a gift from Dr Pier P. Pandolfi (Harvard Medical School, Boston, MA)^59^. The MSCVneo-*MLL-AF9* plasmid^60^ was kindly provided by Dr Scott Armstrong (Harvard Medical School, Boston, MA). The MSCV-*FLT3*-CDS plasmid^61^ was a gift from Dr Michael Cleary (Stanford University, Stanford, CA). The p1005-*Crtc1* plasmid was provided by Dr Sheena Josselyn (The Hospital for Sick Children, Toronto, Canada)^62^, and sub-cloned into MSCV-PIG vector. The MSCV-PIG-*MYCBP* plasmid was PCR-amplified using primers: forward 5′- AAACTCGAGATGGCCCATTACAAAGC -3′ and reverse 5′- CCGGAATTCCTATTCAGCACGC -3′. The 3′UTR constructs of *CRTC1* and *FLT3* containing putative binding sites for miR-22 were amplified by PCR from human normal bone marrow mononuclear cells using the primers below:

*CRTC1*-3′UTR: forward 5′- GCCATTACTAGTCCCACCTGAGTG -3′and reverse 5′- GCCATTAAGCTTGAGGACAGAAGC -3′;

*FLT3*-3′UTR: forward 5′- GCCGCCACTAGTAGGAACAATTTAGTTTTAAGG -3′ and reverse 5′- CGCAAGCTTGTGGGGACAAGAGTAACTTTA -3′, and then cloned into pMIR-REPORT Luciferase miRNA Expression Reporter Vector (Ambion, Austin, TX). Site mutations were induced by PCR based on the sequence shown previously for the miR-22 binding site(s) mutant of 3′UTR of *CRTC1* and *FLT3*. The miR-22 promoter region (−1,100/+55 bp, as was identified by Bar *et al*.^45^) was PCR-amplified using primers: forward 5′-A ATAATGAGCTCAAGGTCGGACG -3′ and reverse 5′- AATAATGATATCCTTTAGCTGGGTC -3′, and cloned into the SacI and EcoRV sites of the pGL4.15 Luciferase Reporter Vector (Promega). The MSCV-*Tet1* construct was as described previously^14^. All the above insertions were confirmed by DNA sequencing.

### Chromatin immunoprecipitation

ChIP assay was performed, as described previously^14^,^17^, with SABiosciences Corporation’s ChampionChiP One-Day kit (Qiagen, Frederick, MD) following the manufacturer’s protocol, with some modifications. Briefly, pellets of 5 × 10^6^ cells were treated with fresh fixing buffer (1% formaldehyde) for 10 min at 37 °C to crosslink DNA and proteins. The reaction was terminated by the addition of stop buffer and incubated at room temperature for 5 min. After cell lysis, the cross-linked chromatin was sonicated to an average size of ∼500 bp and was immunoprecipitated with antibodies against TET1, GFI1 (Santa Cruz Biotechnology Inc., Santa Cruz, CA), the N′-terminal portion of MLL (MLL-N), the C′-terminal of MLL (MLL-C), H3K27Me3, H3K4Me3, RNA polymerase II, EZH2, SIN3A or IgG (Abcam, Cambridge, MA). Purified ChIP DNA was amplified by real-time qPCR using specific primers targeting the CpG-enriched upstream region of human miR-22: forward: 5′- GTTGTTGGAGTCGTGAGTG -3′; reverse: 5′- CGCTCCACCTTTCCTTAAA -3′; or mouse miR-22: forward: 5′- TGAATGGGCGGGAGTAA -3′; reverse: 5′- CCACGAGCTGCGAATGAA -3′.

### Bisulfite sequencing

THP-1 cells were treated with 1 μM ATRA or DMSO control for 72 h. Genomic DNA was extracted thereafter. One microgram of genomic DNA was then applied to MethylCode Bisulfite Conversion Kit (Invitrogen) following the manufacturers’ instructions. After bisulfite conversion, 3 μl of purified converted DNA was PCR-amplified using ZymoTaq DNA polymerase (Zymo Research, Irvine, CA) following the manufacturers’ instructions. The PCR products were purified using PCR purification kits (Qiagen) and sent for sequencing. Primers applied in the PCR assays: miR-22 promoter: forward: 5′- TTTGTTTATTTTTGTTTTTTGGTT -3′; reverse: 5′- ACAACCCCTCCTTATTAAAATC -3′; *SLC43A2:* forward: 5′- TGTTTTGTTTTTATGGAGTGATTTG -3′; reverse: 5′- AAAAATAACCATAAACCATCCTTCC -3′.

### Luciferase reporter and mutagenesis assays

Luciferase reporter and mutagenesis assays were conducted as described previously^17^,^23^, with some modifications. Briefly, for transfection, HEK-293T cells were plated in 96-well plates at a concentration of 6,000 cells per well in triplicate for each condition. For the miR-22 targeting CRTC1 and FLT3 experiments, after overnight incubation, cells were transfected with 20 ng of the pMIR-REPORT bearing the CRTC1 or FLT3 3′UTR or the 3′UTRs with miR-22 binding site mutations, and 20 ng of MSCV-miR-22 or an empty MSCV vector using Effectene Transfection Reagent (Qiagen) according to the manufacturer’s protocol. pMIR-REPORT Beta-galactosidase Reporter Control Vector (Ambion) (1 ng) was co-transfected for transfection efficiency control in all transfections. Cells were lysed and firefly luciferase and β-galactosidase activities were detected using Dual-Light Combined Reporter Gene Assay System (Applied Biosystems, Foster City, CA) 48 h post transfection. Firefly luciferase activity was normalized to β-galactosidase activity for each transfected well. For the Tet1 targeting miR-22 study, HEK-293T cells were transfected with 20 ng MSCV-Tet1 construct and/or 20 ng pGL4.15-miR-22 promoter. The succeeding luciferase reporter assay was conducted according to the manufacture’s protocol (Promega). Each experiment was performed in triplicate and repeated three times.

### Co-immunoprecipitation analysis

For immunoprecipitation, cells were washed with ice-cold PBS and lysed in 800 μl Nonidet P-40 solubilization buffer (50 mM Hepes, pH 8.0, 250 mM NaCl, 0.5% Nonidet P-40, 10% glycerol, 2 mM EDTA, 1 mM NaF, plus 10 μg ml^−1^ aprotinin, 10 μg ml^−1^ benzamidine and 0.2 mM PMSF). The following procedures are performed as described previously^63^. GFI1 was precipitated by using protein A Sepharose beads coated with 400 ng rabbit anti-GFI1 antibody (Santa Cruz Biotechnology Inc.). Lysates and immunoprecipitation complexes were separated and detected by western blotting.

### Packaging of recombinant retroviruses and CFA assays

Those experiments were conducted as described previously^17^,^23^ with some modifications. Briefly, retrovirus vectors were co-transfected with pCL-Eco packaging vector (IMGENEX, San Diego, CA) into HEK-293T cells using Effectene Transfection Reagent (Qiagen) to produce the retroviruses. BM cells were collected from a cohort of 4–6-week-old B6.SJL (CD45.1) donor mice after 5 days of 5-fluorouracil (5-FU) treatment, and primitive hematopoietic progenitor cells were enriched with Mouse Lineage Cell Depletion Kit (Miltenyi Biotec Inc., Auburn, CA). An aliquot of enriched hematopoietic progenitor cells were added to retroviral supernatant together with polybrene in a conical tube, which were centrifuged at 2,000*g* for 2 h at 32 °C (that is, ‘spinoculation’^14^,^17^,^23^) and then the media was replaced with fresh media and incubated for 20 h at 37 °C. Next day, the same procedure was repeated once.

Then, on the day following the second spinoculation, an equivalent of 2.0 × 10^4^ cells were plated into a 35-mm Petri dish in 1.5 ml of Methocult M3230 methylcellulose medium (Stem Cell Technologies Inc., Vancouver, Canada) containing 10 ng ml^−1^ each of murine recombinant IL-3, IL-6, and granulocyte-macrophage colony-stimulating factor (GM-CSF), and 30 ng ml^−1^ of murine recombinant SCF (R&D Systems, Minneapolis, MN), along with 1.0 mg ml^−1^ of G418 and/or 2 μg ml^−1^ of puromycin. For each transduction, there were two duplicate dishes. Cultures were incubated at 37 °C in a humidified atmosphere of 5% CO_2_ in air. The colonies were replated every 7 days under the same conditions. The colony-forming/replating assays were repeated three times.

### Primary and secondary BMT

These experiments were conducted as described previously^14^,^17^,^23^ with some modifications.

For primary BMT assays shown in Fig. 1e, normal bone marrow cells of B6.SJL (CD45.1) mice were retrovirally transduced with MSCV-neo+MSCV-PIG (as control; Ctrl), MSCV-neo+MSCV-PIG-miR-22 (that is, miR-22), MSCV-neo-*MLL-AF9*+MSCV-PIG (that is, MA9), MSCV-neo-*MLL-AF9*+MSCV-PIG-miR-22 (that is, MA9+miR-22) or MSCV-neo-*MLL-AF9*+MSCV-PIG-miR-22 mutant (that is, MA9+miR-22mut), through two rounds of spinoculation. Then, retrovirally transduced cells were plated into methylcellulose medium supplied with a set of cytokines to form colonies as described in the CFA assays. Seven days later, colony cells were collected and washed, and then were injected by tail vein into lethally irradiated (960 rads) 8–10-week-old C57BL/6 (CD45.2) recipient mice with 1.5 × 10^5^ donor cells plus a radioprotective dose of whole BM cells (1 × 10^6^; freshly collected from a C57BL/6 mouse) per recipient mouse. Notably, as the colony cells were under selection of both G418 (1.0 mg ml^−1^) and puromycin (2 μg ml^−1^) for a week, all donor cells (that is, the collected colony cells) must be positive for retroviral transductions of both MSCVneo- and MSCV-PIG-based constructs. Thus, *MLL-AF9* and miR-22 (or miR-22mut) must be ectopically co-expressed in MA9+miR-22 (or MA9+miR-22mut) donor cells, which actually were confirmed by qPCR. Indeed, due to the potent inhibitory effect of miR-22 on MLL-AF9-induced colony forming, we had to prepare more mouse BM progenitor cells for the co-transduction of MLL-AF9 and miR-22. Thus, we plated them in a larger number of dishes than what we did for other groups of co-transductions. After BMT, all recipient mice were watched for leukemogenesis for a period of 200 days or till the end point that the mice developed full-blown AML or other severe illness.

For primary BMT assays shown in Fig. 1g, C57BL/6 mouse (CD45.2) BM progenitor cells were co-transduced with MSCVneo-*MLL-AF10*, together with MSCV-PIG-miR-22 or MSCV-PIG vector. Cells were grown in RPMI medium 1640 (Invitrogen) containing 10% FBS, 1% HEPES and 1% penicillin–streptomycin, supplemented with a supply of SCF, IL-3 and IL-8. The cells were selected with both puromycin and G418 for 7 days before transplantation. After that, 1.5 × 10^5^ donor cells plus a radioprotective dose of whole bone marrow cells (1 × 10^6^; freshly collected from a B6.SJL (CD45.1) mouse) were injected into per lethally irradiated (960 rads) 8–10-week-old B6.SJL recipient mouse.

For primary BMT assays shown in Fig. 1h, C57BL/6 mouse (CD45.2) BM progenitor cells or miR-22^−/−^ BM progenitor cells were retrovirally transduced with MSCV-PIG-AE9a. The cells were selected with puromycin for 7 days before transplantation. After that, 1.5 × 10^5^ donor cells plus a radioprotective dose of whole bone marrow cells (1 × 10^6^; freshly collected from a B6.SJL (CD45.1) mouse) were injected into per lethally irradiated (960 rads) 8–10-week-old B6.SJL recipient mouse.

For secondary BMT assay shown in Fig. 2a,c,d, leukaemic BM cells isolated from the primary leukaemic mice bearing *MLL-AF9*, *AML1-ET9a* (*AE9a*) or *FLT3*-ITD/*NPM1*c^+^ were retrovirally transduced with MSCV-PIG+MSCVneo (as control; MA9-AML+Ctrl, AE9a-AML+Ctrl or *FLT3*-ITD/*NPM1*c^+^-AML+Ctrl) or MSCV-PIG+MSCVneo-miR-22 (that is, MA9-AML+miR-22, AE9a-AML+miR-22, or *FLT3*-ITD/*NPM1*c^+^-AML+miR-22). Similarly, retrovirally transduced cells were plated into methylcellulose medium supplied with puromycin and G418 (for selection) and a set of cytokines to form colonies. Seven days later, the colony cells were collected and washed, and then were transplanted into sub-lethally irradiated (480 rads) 8–10-week-old C57BL/6 (CD45.2) secondary recipient mice via tail vein injection, with the dosage of 1.5 × 10^5^ donor cells per recipient mouse.

For secondary BMT assays shown in Fig. 4f, leukaemic BM cells isolated from the primary leukaemic mice bearing *MLL-AF9* fusion were retrovirally transduced with MSCV-neo+MSCV-PIG (that is, MA9-AML+Ctrl), MSCV-neo-miR-22+ MSCV-PIG (that is, MA9-AML+miR-22), MSCV-neo-miR-22+MSCV-PIG-*CRTC1* (that is, MA9-AML+miR-22+CRTC1), MSCV-neo-miR-22+MSCV-PIG-*FLT3* (that is, MA9-AML+miR-22+FLT3) or MSCV-neo-miR-22+MSCV-PIG-*MYCBP* (that is, MA9-AML+miR-22+MYCBP). Again, retrovirally transduced cells were plated into methylcellulose medium supplied with G418 and puromycin (for selection) as well as a set of cytokines to form colonies. Seven days later, the colony cells were collected and washed, and then were transplanted into sub-lethally irradiated (480 rads) 8–10-week-old C57BL/6 (CD45.2) secondary recipient mice via tail vein injection, with the dosage of 1.5 × 10^5^ donor cells per recipient mouse.

### Preparation of Cy5.5-labelled G7 PAMAM dendrimers

G7 PAMAM dendrimers obtained from Sigma-Aldrich were purified and fluorescently labelled using an *N*-hydroxysuccinimide ester of cyanine5.5 (NHS-Cy5.5) (Lumiprobe Corporation, Hallandale Beach, FL), as has been previously reported^64^. In brief, G7 PAMAM dendrimers (38.7 mg, 332 nmol) were dissolved in 2 ml ddH_2_O, to which NHS-Cy5.5 (3.75 mg, 3.32 μmol) in 400 μl DMSO was added dropwise, and the reaction allowed to proceed under vigorous stirring for 24 h at room temperature. Excess NHS-Cy5.5 was removed using an Amicon Ultra-15 Centrifugal Filter Unit (MWCO 10,000, Millipore, Billerica, MA) at 4,000 r.p.m. and 4 °C for 20 min and washing with ddH_2_O 10 times. Remaining product was re-dissolved in ddH_2_O and lyophilized, resulting in G7-Cy5.5-NH_2_. All products were characterized by ^1^H NMR using a 400-MHz Bruker DPX-400 spectrometer (Bruker BioSpin Corp., Billerica, MA).

### Nanoparticle treatment in BMT or xeno-transplantation models

For secondary BMT followed with G7-Cy5.5-NH_2_ dendrimer treatment shown in Fig. 7a,b, leukaemic BM cells isolated from the primary leukaemic mice (CD45.1) bearing *MLL-AF9* or *AE9a* fusion were transplanted into sub-lethally irradiated (480 rads) 8–10-week-old C57BL/6 (CD45.2) secondary recipient mice via tail vein injection, with the dosage of 1.5 × 10^5^ donor cells per recipient mouse. After the onset of leukaemia (when mice had an engraftment (CD45.1) over 20% and/or white blood cell counts higher than 4 × 10^9^ l^−1^; for the *MLL-AF9* and *AE9a* secondary transplantation models, usually 10 days post transplantation), the recipient mice were injected with PBS control, or 0.5 mg kg^−1^ miR-22 or miR-22 mutant RNA oligos formulated with G7-NH2 nanoparticles, i.v., every other day, until the PBS-treated group all died of leukaemia.

For xeno-transplantation followed with G7-Cy5.5-NH2 dendrimer treatment shown in Fig. 7c, MV4;11 cells were transplanted into NSGS (NSG-SGM3) mice via tail vein injection, with the dosage of 5 × 10^5^ donor cells per recipient mouse. Five days after xeno-BMT, the recipient mice were injected with PBS control, or 0.5 mg kg^−1^ miR-22 or miR-22 mutant RNA oligos formulated with G7-NH2-nanoparticles, i.v., every other day, until the PBS-treated mice all died of leukaemia.

### The maintenance and monitoring of mice

C57BL/6 (CD45.2), B6.SJL (CD45.1) mice were purchased from the Jackson Lab (Bar Harbor, ME, USA) or Harlan Laboratories, Inc (Indianapolis, IN, USA). NSGS (NSG-SGM3) immunodeficient mice^49^ and miR-22^−/−^ (ref. 20) mice were purchased from the Jackson Lab and were bred and maintained in house. Both male and female mice were used for the experiments. All laboratory mice were maintained in the animal facility at the University of Chicago and the University of Cincinnati. All experiments on mice in our research protocol were approved by Institutional Animal Care and Use Committee (IACUC) of the University of Chicago and the University of Cincinnati. The maintenance, monitoring and end-point treatment of mice were conducted as described previously^14^,^17^,^23^.

### Western blotting

Western blotting was conducted as described previously^14^,^17^,^23^ with some modifications. Briefly, transiently transfected MONOMAC-6 cells were collected and lysed with RIPA buffer (Thermo Scientific, BufferRockford, IL). Proteins from the lysate were fractionated by electrophoresis through 4–15% polyacrylamide gels (Bio-rad, Hercules, CA) and transferred to polyvinylidene fluoride membranes using tris-glycine transfer buffer (Thermo Scientific). Blots were incubated with IRDye 800CW-conjugated or 700CW-conjugated antibody and infrared fluorescence images were obtained with the Odyssey infrared imaging system (Li-Cor Bioscience, Lincoln, NE). 100-200 ng ml^−1^ anti-CRTC1, anti-FLT3, anti-MYCBP, anti-BMI1, anti-CDK6, anti-PGK1 (Santa Cruz Biotechnology Inc.), anti-HMGA1 (Abcam) and anti-GAPDH (Thermo Scientific) antibodies were used to detect corresponding proteins. Original scans can be found as Supplementary Figures 7–9.

### Target gene prediction

Putative targets of miR-22 was predicted by TargetScan ( http://www.targetscan.org)^18^.

### DNA copy-number analysis of miR-22 gene locus in human AML

The copy-number data of AML from The Cancer Genome Atlas (TCGA) project were downloaded from Broad Firehose’s analyses runs. The putative copy-number calls were determined using GISTIC 2.0 (ref. 65). The latest GISTIC analyses data were obtained using the following shell command: ‘firehose_get -o ‘GISTIC’ analyses latest LAML’.

The.cel files of Affymetrix SNP 6.0 data for GSE21107 (ref. 66) and GSE23452 (ref. 67) were downloaded from NCBI GEO. The raw data were preprocessed using PennCNV^68^. Then ASCAT^69^ was used to obtain the copy-number alterations. The putative copy-number calls were determined using GISTIC 2.0 as described above.

### Software and statistical analyses

The miRNA and gene/exon array data analyses, as well as qPCR data analyses were conducted by use of Partek Genomics Suite (Partek Inc.), TIGR Mutiple Array Viewer software package (TMeV version 4.6; TIGR, Rockville, MA)^70^ and/or Bioconductor R packages. The miRNA-gene expression correlation was analysed by use of Partek Genomics Suite (Partek Inc.). The *t*-test, Kaplan–Meier method and log-rank testand so on were performed with WinSTAT (R. Fitch Software), GraphPad Prism version 5.00 (GraphPad Software, San Diego, CA) and/or Partek Genomics Suite (Partek Inc.). The *P* values <0.05 were considered as statistically significant. Significance analysis of microarrays, embedded in the TMeV package (TIGR, Rockville, MA), was used to identify the genes that are significantly (*q*<0.05; false discovery rate, FDR<0.05) dysregulated in the *MLL-AF9*-mediated mouse leukaemia samples or human AML samples relative to the normal controls. Pearson correlation was used in the analysis of the correlation between miR-22 and candidate genes in expression. The list of transcription factors that have evolutionarily conserved binding sites within the miR-22 promoter region (that is, the adjacent upstream CpG island) was obtained by searching UCSC Genome Browser ( https://genome.ucsc.edu/cgi-bin/hgTracks?db=hg19&position=chr17%3A1614689-1623188&hgsid=467686877_3vyTlry3a40ZiT7dfAaAIAsYA2R6).

### Data availability

Data referenced in this study are available in The Gene Expression Omnibus. The Affymetrix exon array data and the microarray data are available under accession codes GSE34184 and GSE30285. Additional exon array data are available under accession code GSE27370. The mouse microarray data is available under accession code GSE34185. The AML samples collected by the German AMLCG study group are available under accession code GSE37642. The mRNA gene expression data of 183 adult *de novo* AML cases is available from the TCGA.

## Additional information

**Accession codes:** The microarray data have been deposited in the Gene Expression Omnibus (GEO) database under the accession code GSE34184, GSE30285, GSE27370, GSE34185, and GSE37642.

**How to cite this article:** Jiang, X. *et al*. miR-22 has a potent anti-tumour role with therapeutic potential in acute myeloid leukaemia. *Nat. Commun.* 7:11452 doi: 10.1038/ncomms11452 (2016).

## Supplementary Material

Supplementary Information Supplementary Figures 1-9, Supplementary Tables 1-4 and Supplementary References.
