# Supplementary material for: miR-22 has a potent anti-tumour role with therapeutic potential in acute myeloid leukaemia
Source: Nat Commun. 2016 Apr 26;7:11452. doi: 10.1038/ncomms11452 (PMC5477496; doi:10.1038/ncomms11452)
Supplement: Supplementary Information — Supplementary Figures 1-9, Supplementary Tables 1-4 and Supplementary References. [file ncomms11452-s1.pdf]

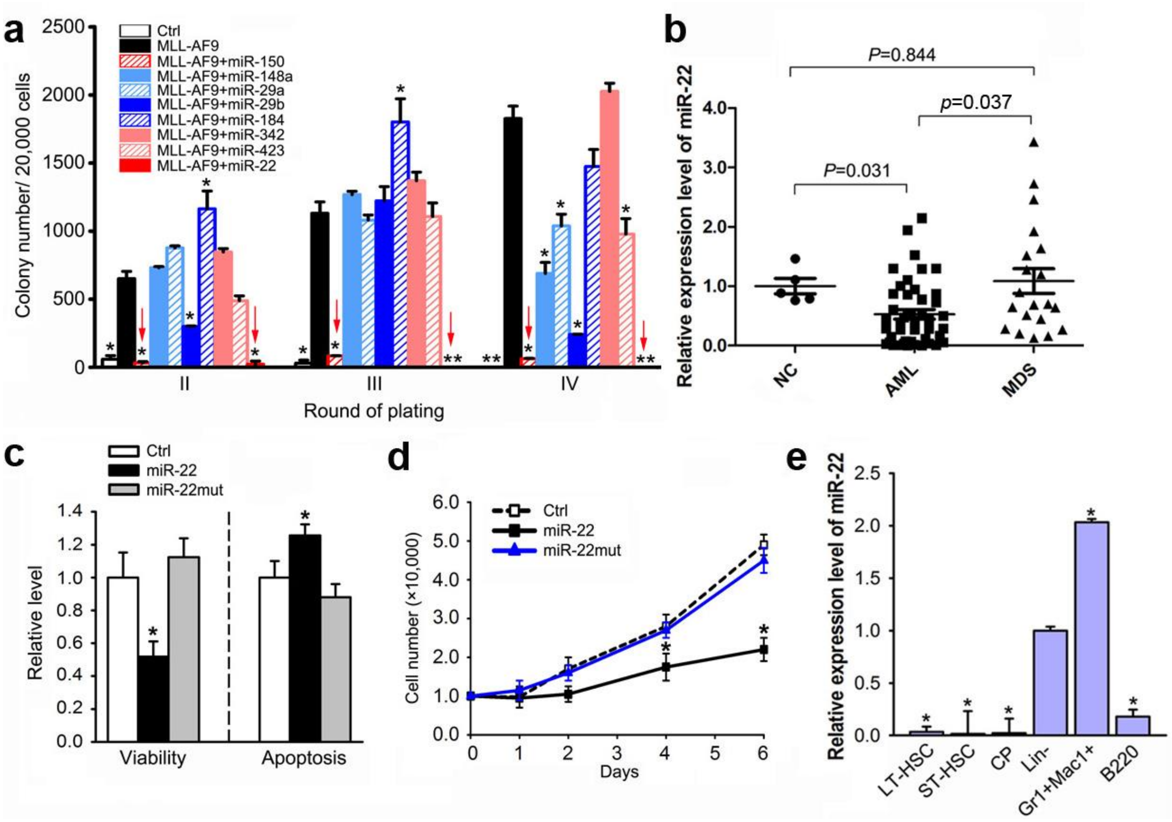

Supplementary Figure 1. miR-22, which is down-regulated in AML, inhibits AML cell transformation and growth. (**a**) Comparison of effects of a set of miRNAs, which are down-regulated in AML, on *MLL-AF9*-induced colony forming. Colony-forming/replating assays (CFAs) were performed using mouse BM progenitor (lineage negative; Lin^-) cells transduced with retrovirus of MSCV-neo+MSCV-PIG (Ctrl), MSCV-neo-*MLL-AF9*+MSCV-PIG (MLL-AF9), or MSCV-neo-*MLL-AF9*+MSCV-PIG-miR-150/miR-148a/miR-29a/miR-29b/miR-184/miR-342/miR-423/miR-22. (**b**) qPCR confirmation of the down-regulation of miR-22 in AML. Expression level of miR-22 in the mononuclear cells of 5 normal MNC control (NC), 42 AML (including 17 *MLL*-rearranged AML, 13 t(8;21), 5 t(15;17) and 7 inv(16)) and 20 MDS (including 17 (+8) and 3 (-7)) samples are shown. (**c, d**) Effects of miR-22 on the viability and apoptosis (**c**) as well as growth (**d**) of AML cells. MONOMAC-6 cells were transfected with MSCV-PIG (Ctrl), MSCV-PIG-miR-22 (miR-22), or miR-22 mutant (miR-22mut) plasmids. Cell viability and apoptosis were detected 48 hrs post-transfection. Cell growth was assessed by cell counts at a series of time points post-transfection. (**e**) Expression levels of miR-22 in various sub-populations of mouse normal hematopoietic cells. Expression levels of miR-22 in long-term HSC (LT-HSC; $Lin^-Sca1^+c\text{-}Kit^+Flk2^-$, $LSKF^-$), short-term HSC (ST-HSC; $Lin^-Sca1^+c\text{-}Kit^+Flk2^+$, $LSKF^+$), committed progenitor (CP; $Lin^-Sca1^-c\text{-}Kit^+$), $Gr\text{-}1^+/Mac\text{-}1^+$ myeloid and $B220^+$ lymphoid cells, were normalized to the level in lineage negative (Lin^-) cells. Mean±SD values are shown. *, $p<0.05$. All the *p* values shown in this figure were generated by *t*-test.

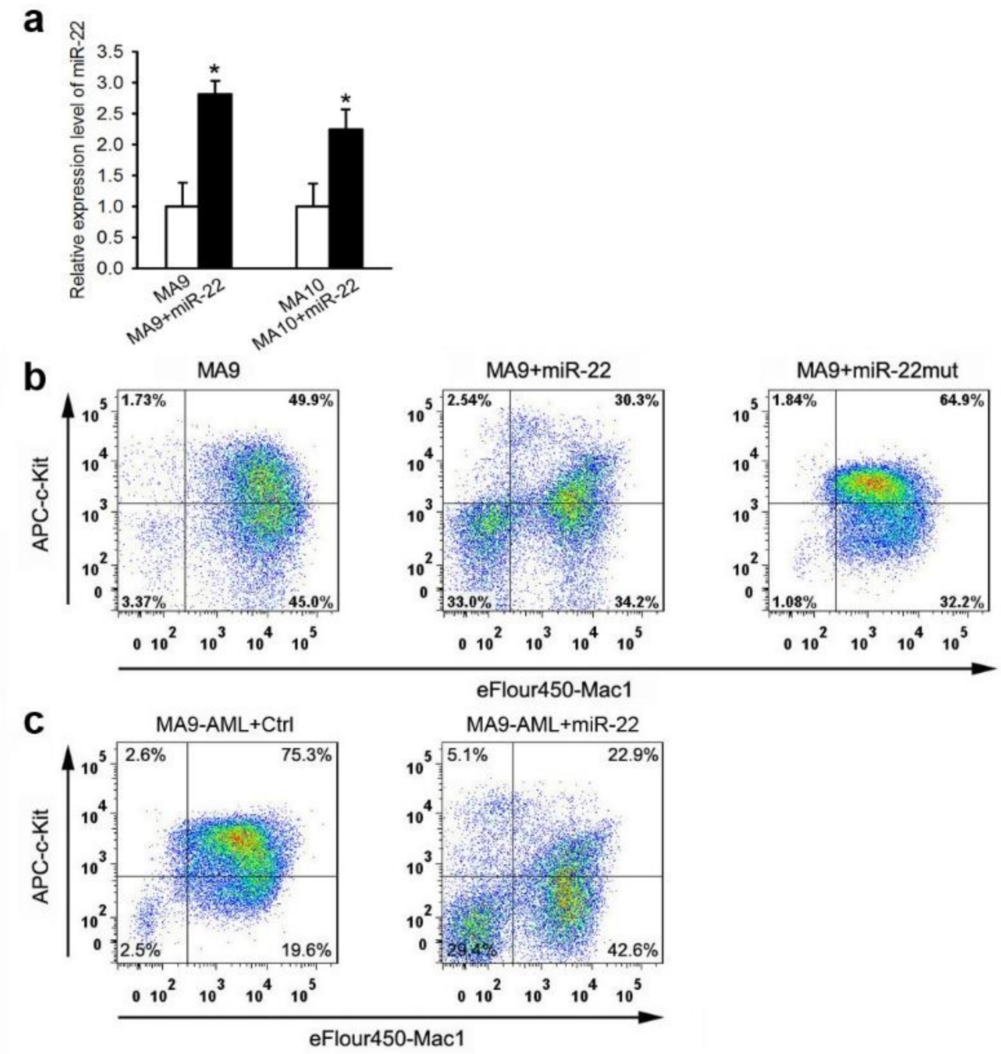

Supplementary Figure 2. Role of miR-22 in AML *in vivo*. (**a**) Overexpression level of miR-22 in bone marrow cells of MLL-AF9 and MLL-AF10-induced primary leukemic mice. 3~5 independent leukemic mice are included in each group. Mean±SD values are shown. *, $p<0.05$, *t*-test. (**b, c**) Flow cytometry analyses of the BM cells of both primary bone marrow transplantation (BMT) recipient mice (**b**) or secondary BMT recipient mice (**c**). 0.5 million mouse BM cells collected at the end point were stained with APC-conjugated anti-mouse c-Kit or eFluor®450-conjugated anti-mouse CD11b (Mac-1) antibodies. Proportions of c-Kit$^+$ and/or Mac1$^+$ cells are shown.

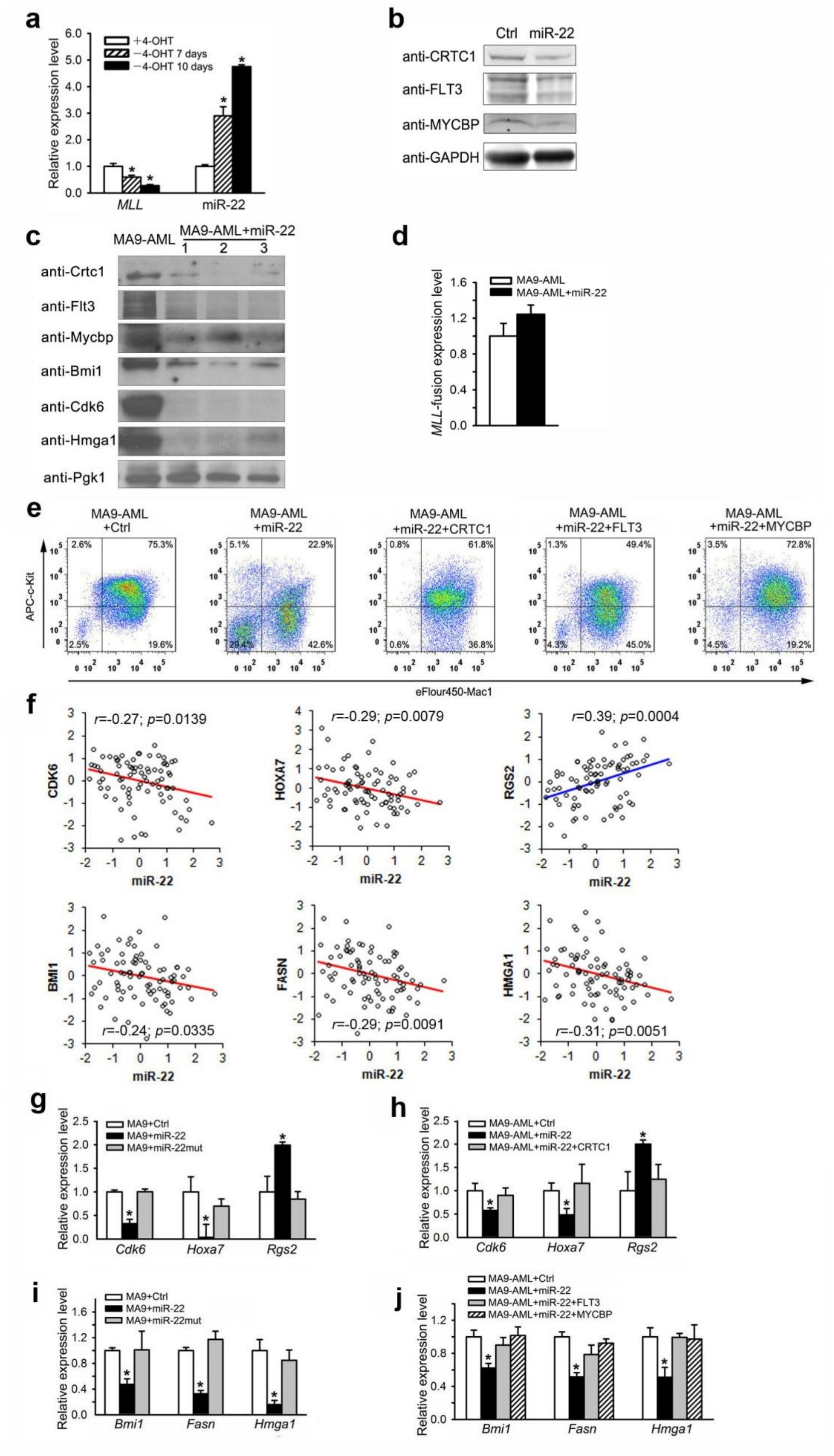
a
Relative expression level
+4-OHT
−4-OHT 7 days
−4-OHT 10 days
MLL
miR-22
b
Ctrl
miR-22
anti-CRTC1
anti-FLT3
anti-MYCBP
anti-GAPDH
c
MA9-AML
MA9-AML+miR-22
1
2
3
anti-Crtc1
anti-Flt3
anti-Mycbp
anti-Bmi1
anti-Cdk6
anti-Hmga1
anti-Pgk1
d
MLL-fusion expression level
MA9-AML
MA9-AML+miR-22
e
MA9-AML +Ctrl
MA9-AML +miR-22
MA9-AML +miR-22+CRTC1
MA9-AML +miR-22+FLT3
MA9-AML +miR-22+MYCBP
APC-c-Kit
eFlour450-Mac1
2.6%
75.3%
2.5%
19.6%
5.1%
22.9%
29.4%
42.6%
0.8%
61.8%
0.6%
36.8%
1.3%
49.4%
4.3%
45.0%
3.5%
72.8%
4.5%
19.2%
f
r=-0.27; p=0.0139
r=-0.29; p=0.0079
r=0.39; p=0.0004
CDK6
HOXA7
RGS2
BMI1
FASN
HMGA1
miR-22
r=-0.24; p=0.0335
r=-0.29; p=0.0091
r=-0.31; p=0.0051
g
Relative expression level
MA9+Ctrl
MA9+miR-22
MA9+miR-22mut
Cdk6
Hoxa7
Rgs2
h
MA9-AML+Ctrl
MA9-AML+miR-22
MA9-AML+miR-22+CRTC1
i
MA9+Ctrl
MA9+miR-22
MA9+miR-22mut
Bmi1
Fasn
Hmga1
j
MA9-AML+Ctrl
MA9-AML+miR-22
MA9-AML+miR-22+FLT3
MA9-AML+miR-22+MYCBP

Supplementary Figure 3. Repression of the CREB and MYC pathways by miR-22. (**a**) Up-regulation of miR-22 level in *MLL-ENL*-ERtm cells after withdrawal of 4-OHT for 0, 7 or 10 days. (**b**) Down-regulation of CRTC1, FLT3 and MYCBP at the protein level by miR-22. MONOMAC-6 cells were transfected with MSCV-PIG-miR-22 (miR-22) or MSCV-PIG vector (Ctrl). Protein extractions of the cells were analyzed through Western-blotting assays 48 hrs post-transfection. (**c**) Down-regulation of Crtc1, Flt3, Mycbp, Bmi1, Cdk6 and Hmga1 by miR-22 overexpression. Protein levels in BM blast cells of leukemic mice transplanted with *MLL-AF9* or *MLL-AF9*+miR-22 primary leukemic cells are shown. (**d**) miR-22 overexpression showed no significant influence on the level of *MLL*-fusion in *MLL-AF9*-leukemic mouse BM cells. Mouse BM progenitor cells bearing MLL-AF9 were retrovirally transduced with MSCV-PIG-miR-22 or MSCV-PIG vector. After 7 days' selection with puromycin, *MLL* levels were detected through qPCR. (**e**) Flow cytometry analyses of the BM cells of secondary BMT mice**.** 0.5 million mouse BM cells collected at the end point were stained with APC-conjugated anti-mouse c-Kit or eFluor®450-conjugated anti-mouse CD11b (Mac-1) antibodies. Proportions of c-Kit$^+$ and/or Mac1$^+$ cells are shown. (**f**) Correlation between the expression levels of miR-22 and *CDK6, HOXA7, RGS2, BMI1, FASN* or *HMGA1* in In-house_81S. The correlation coefficient (*r*) and *p* values were detected by "Pearson Correlation", and the correlation regression lines were drawn with the "linear regression" algorithm. (**g-j**) Expression levels of the CREB downstream genes *Cdk6, Hoxa7* and *Rgs2,* as well as the MYC downstream genes *Bmi1, Fasn* and *Hmga1* were detected in the BM cells of the primary BMT recipient mice (**g, i**) or the secondary BMT recipients (**h, j**). The primary transplanted mice include *MLL-AF9*+Ctrl, *MLL-AF9*+miR-22, and *MLL-AF9*+miR-22 mutant (miR-22mut). The secondary recipients were transplanted with the blast cells of the primary *MLL-AF9* mice retrovirally transduced with MSCVneo+MSCV-PIG (MA9-AML+Ctrl), MSCVneo-miR-22+MSCV-PIG (MA9-AML+miR-22), or MSCVneo-miR-22+MSCV-PIG-*CRTC1/FLT3/MYCBP* (MA9-AML+miR-22+CRTC1/FLT3/MYCBP). n=5 for each group. Mean±SD values of three replicates are shown. *, $p<0.05$. All the *p* values shown in this figure were generated by *t*-test, except for those shown in Figure 3F that were generated by Pearson correlation test.

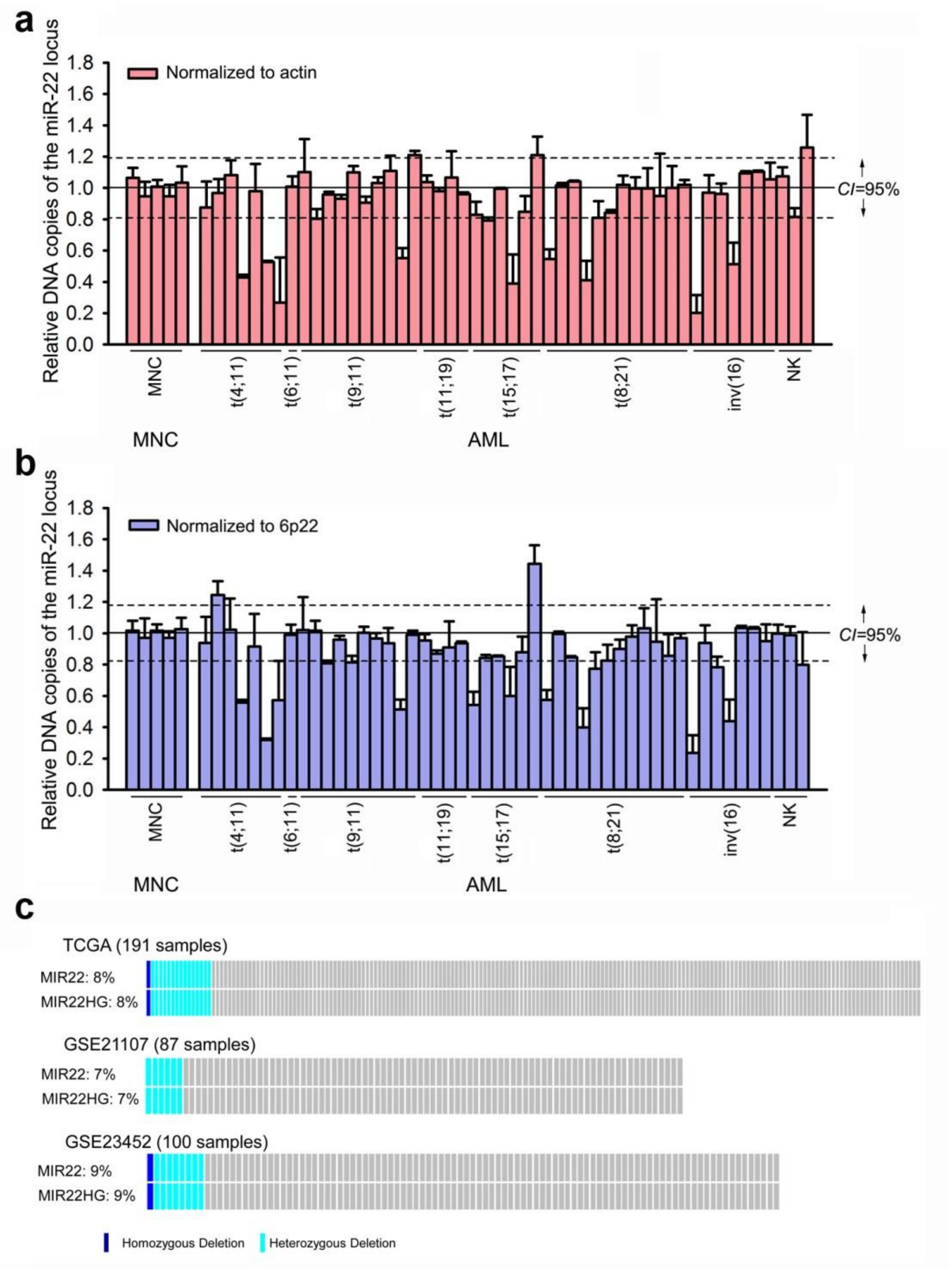

Supplementary Figure 4. DNA copy-number loss of the miR-22 gene locus in AML. (**a, b**) DNA copy number of the miR-22 gene locus of 50 AML samples and 5 normal controls were tested. qPCR data were normalized against the chromosomal region of *actin* (**a**) or *6p22* (**b**) probe. Mean values, standard deviations (error bars), and the 95% confidence internal (*CI*) are shown. (**c**) DNA copy-number loss of miR-22 in three independent AML datasets are shown. *Dark blue*: Homozygous deletion; *Cyan*: Heterozygous deletion.

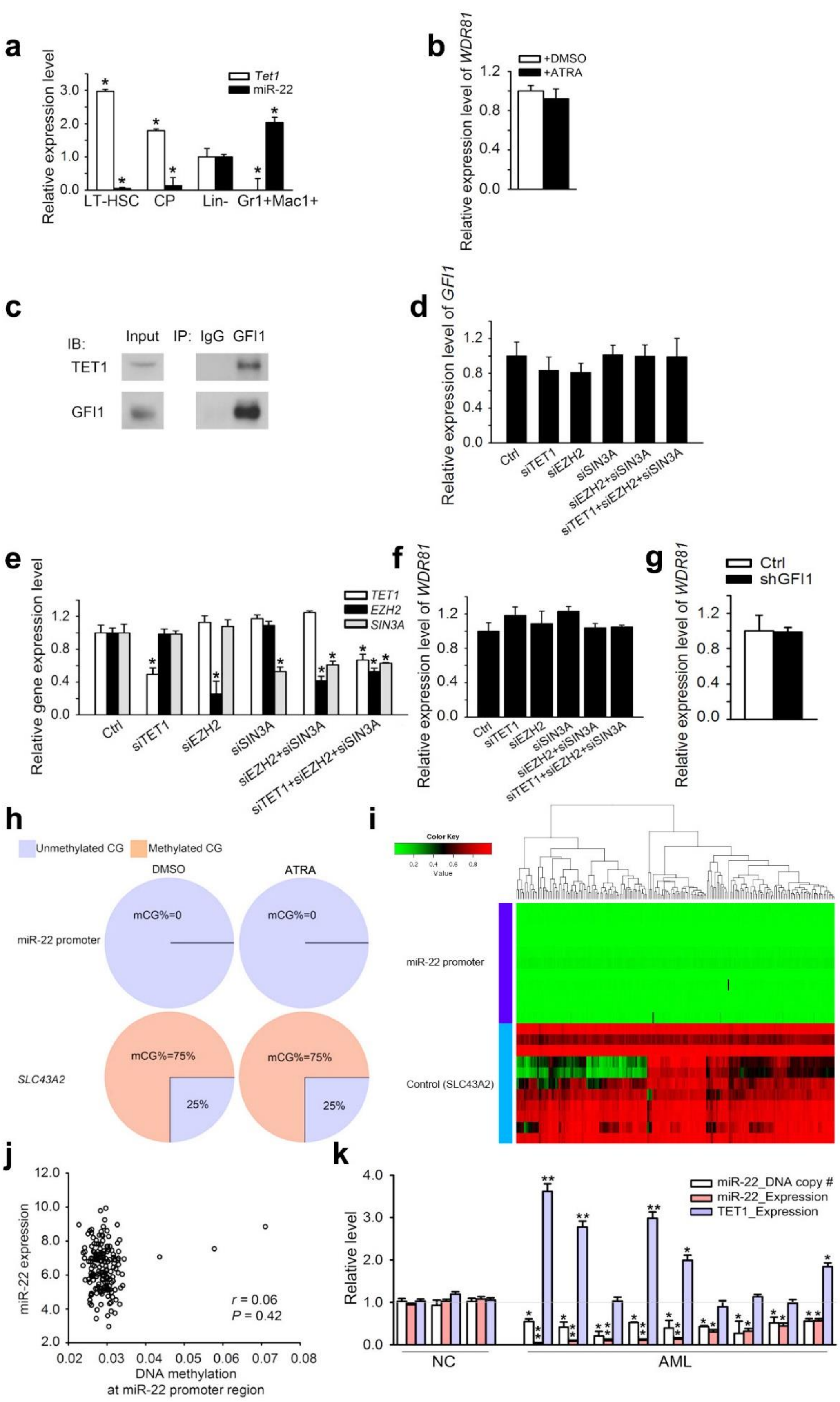
a
Relative expression level
Tet1
miR-22
LT-HSC
CP
Lin-
Gr1+Mac1+
b
Relative expression level of WDR81
+DMSO
+ATRA
c
IB:
Input
IP:
IgG
GFI1
TET1
GFI1
d
Relative expression level of GFI1
Ctrl
siTET1
siEZH2
siSIN3A
siEZH2+siSIN3A
siTET1+siEZH2+siSIN3A
e
Relative gene expression level
TET1
EZH2
SIN3A
f
Relative expression level of WDR81
g
Relative expression level of WDR81
Ctrl
shGFI1
h
Unmethylated CG
Methylated CG
DMSO
ATRA
miR-22 promoter
mCG%=0
SLC43A2
mCG%=75%
25%
i
Color Key
Value
miR-22 promoter
Control (SLC43A2)
j
miR-22 expression
DNA methylation
at miR-22 promoter region
r = 0.06
P = 0.42
k
Relative level
miR-22_DNA copy #
miR-22_Expression
TET1_Expression
NC
AML

Supplementary Figure 5. Involvement of TET1 in miR-22 regulation. (**a**) Expression levels of Tet1 and miR-22 in various sub-populations of mouse normal hematopoietic cells. Gene expression levels in long-term HSC (LT-HSC; $Lin^{-}Sca1^{+}c\text{-}Kit^{+}Flk2^{-}$, $LSKF^{-}$), committed progenitor (CP; $Lin^{-}Sca1^{-}c\text{-}Kit^{+}$) and $Gr\text{-}1^{+}/Mac\text{-}1^{+}$ myeloid cells, were normalized to the level in lineage negative (Lin^{-}) cells. (**b**) Expression of *WDR81* in THP-1 cells 72 hours post-treatment with 1 μM ATRA or DMSO control. (**c**) Co-immunoprecipitation showing the binding between GFI1 and TET1 in HEK293T cells. (**d**) Effects of knockdown of *TET1*, *EZH2* and/or *SIN3A* on *GFI1* expression. (**e**) siRNA knockdown effects on *TET1*, *EZH2* and *SIN3A* in THP-1 cells. Gene levels were detected 48 hrs post-transfection. (**f**) Effects of knockdown of *TET1*, *EZH2* and/or *SIN3A* on *WDR81* expression. The expression level of *WDR81*was detected in THP-1 cells 72 hours post-transfection with siRNAs targeting *TET1*, *EZH2* and/or *SIN3A*. (**g**) Effects of knockdown of *GFI1* on *WDR81* expression. The expression level of *WDR81* was detected in THP-1 cells transduced with lentiviral vectored *GFI1* shRNA or control shRNA. (**h**) DNA methylation at miR-22 promoter region determined by bisulfite sequencing. Percentage of methylated CG (mCG%) at miR-22 promoter region in THP-1 cells treated with DMSO or 1 μM ATRA for 72 hrs were analyzed, with *SLC43A2* (100 kbps downstream of miR-22 promoter) as a positive control of DNA methylation. (**i**) DNA methylation level at miR-22 promoter region of 194 AML patient samples from TCGA_194S is shown, with *SLC43A2* as a positive control of DNA methylation. (**j**) Correlation analysis between miR-22 methylation and expression in 170 AML patient samples with both methylation and gene expression data from TCGA_194S. (**k**) qPCR analysis of miR-22 and *TET1* expression in AML samples with DNA copy-number loss of miR-22. A total of 9 AML and 3 normal controls samples, all been studied in the analyses of Supplementary Figure 4a/b, are included. The expression levels of miR-22 and TET1 are normalized to *RNU48* and *actin*, respectively. The DNA copy numbers are normalized to those at the gene locus of *actin*. Mean±SD values are shown. *, $p<0.05$; **, $p<0.01$. All the p values shown in this figure were generated by t-test, except for the one shown in Figure 5J that was generated by Pearson correlation test.

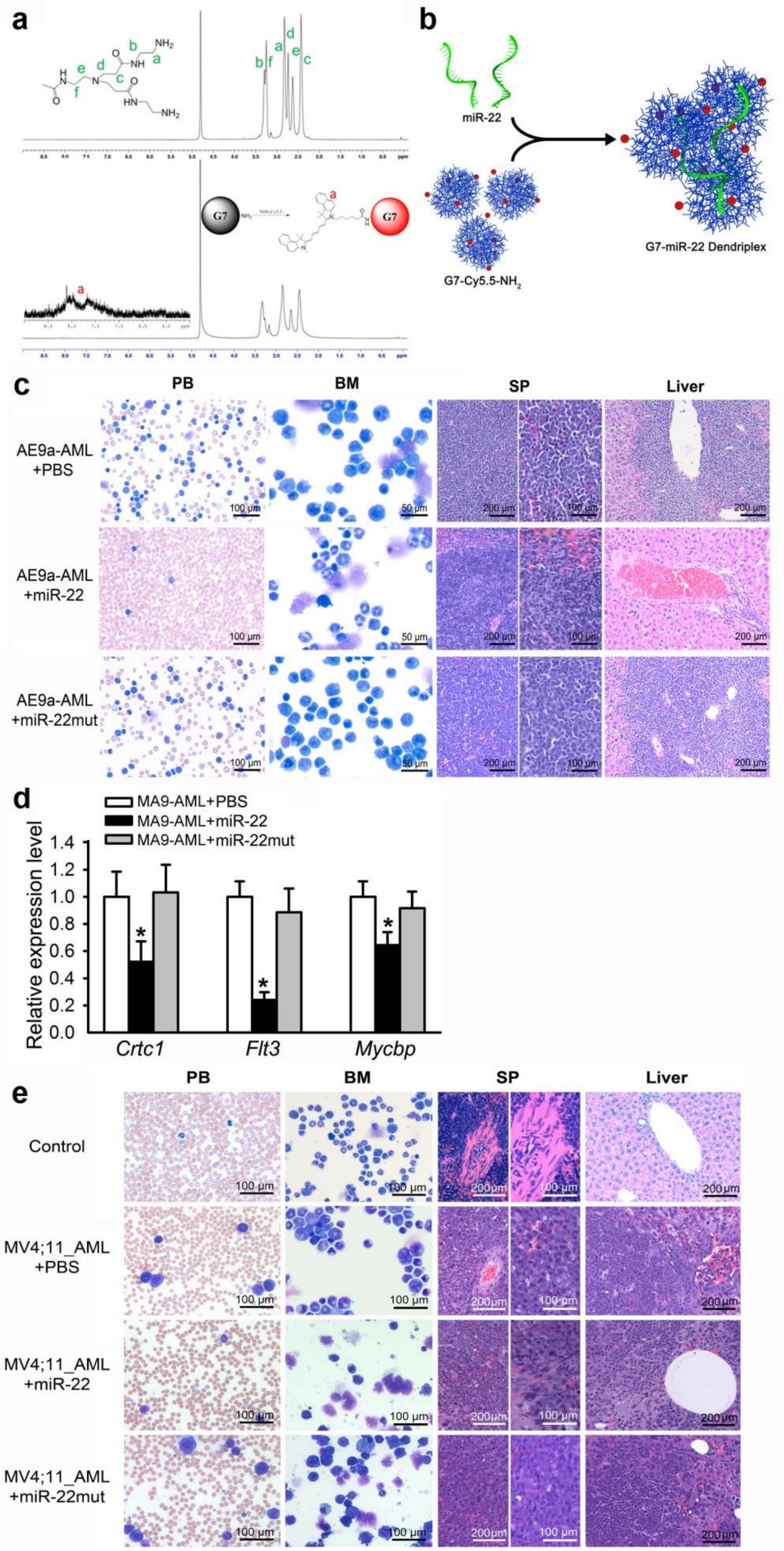
a
b
miR-22
G7-Cy5.5-NH2
G7-miR-22 Dendriplex
c
PB
BM
SP
Liver
AE9a-AML
+PBS
AE9a-AML
+miR-22
AE9a-AML
+miR-22mut
d
MA9-AML+PBS
MA9-AML+miR-22
MA9-AML+miR-22mut
Relative expression level
Crtc1
Flt3
Mycbp
e
Control
MV4;11_AML
+PBS
MV4;11_AML
+miR-22
MV4;11_AML
+miR-22mut

Supplementary Figure 6. The formation and effects of G7-Cy5.5-NH_2-miR-22 nanoparticles. (**a**) ^{1}H NMR spectra of G7 PAMAM dendrimer (*upper panel*) and G7-Cy5.5-NH_2 (*lower panel*). Characteristic dendrimer peaks are located between 2.0–3.5 ppm. Following conjugation, there is an appearance of new peaks between 7.0-8.5 ppm due to the aromatic protons on Cy5.5, corresponding to approximately 4 Cy5.5 per G7. (**b**) Formation of the G7-miR-22 dendriplex. G7 PAMAM dendrimers were labeled using the near infrared dye, Cy5.5 (red dots), and mixed with miR-22. Complex formation is driven by electrostatic forces between the cationic primary amine surface groups of the PAMAM dendrimer and the anionic phosphate backbone of miR-22. Schematic represents approximate theoretical mixing ratio and sizes of polyplex components. (**c**) Wright-Giemsa stained PB and BM, and H&E stained spleen and liver of the *AE9a*-secondary leukemic mice treated with PBS control, miR-22 or miR-22 mutant nanoparticles. (**d**) Expression levels of *Crtc1, Flt3* and *Mycbp* in the BM cells of the PBS, miR-22- or miR-22 mutant-nanoparticle-treated secondary BMT recipient mice transplanted with *MLL-AF9* primary leukemic blast cells. Mean±SD values are shown. *, $p<0.05$, *t*-test. (**e**) Wright-Giemsa stained PB and BM, and H&E stained spleen and liver of the non-transplanted NSGS control or MV4;11 xenotransplanted NSGS leukemic mice treated with PBS control, miR-22 or miR-22 mutant nanoparticles.

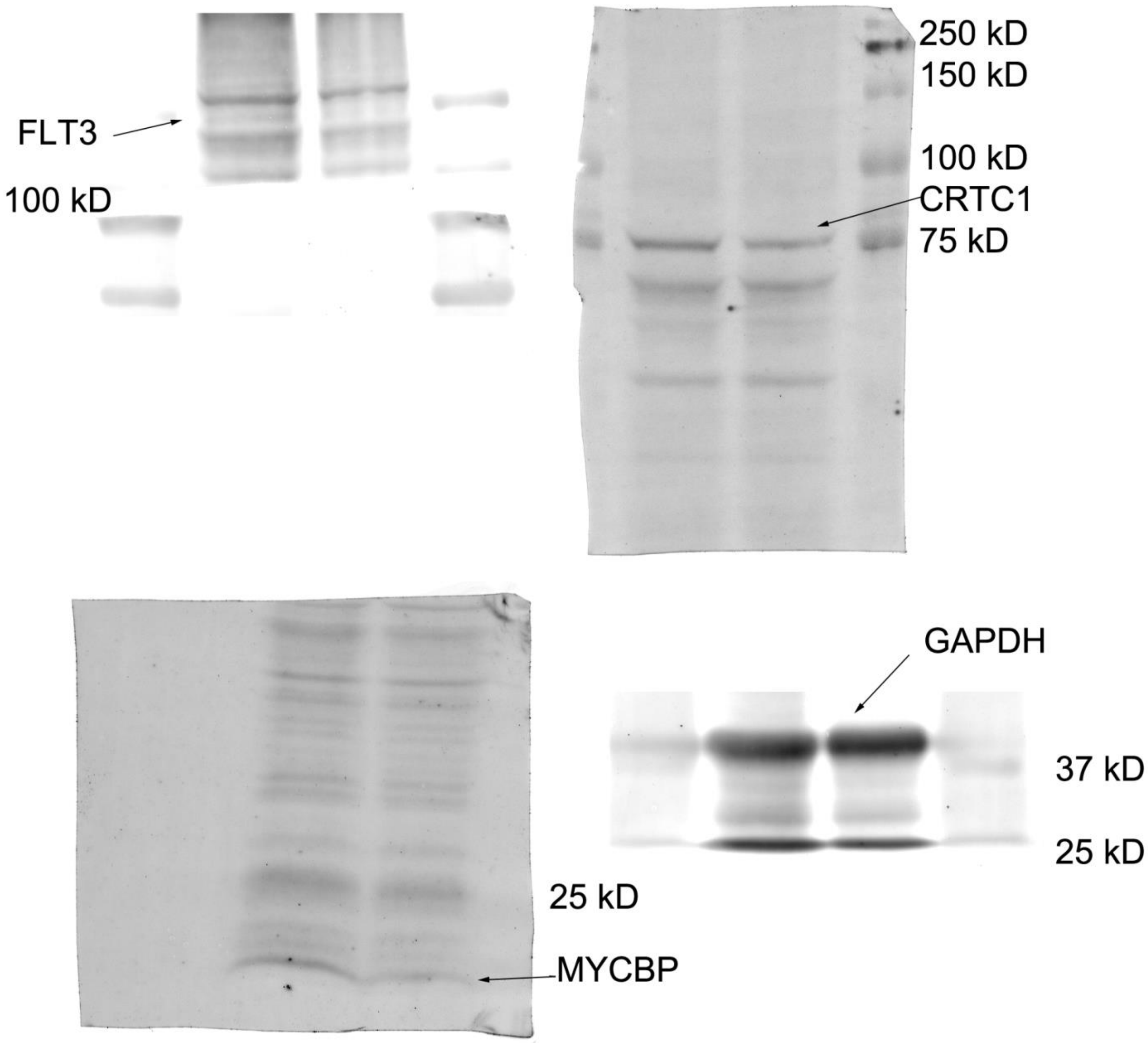

Supplementary Figure 7. Original scans of Western blotting results shown as Supplementary Figure 3b.

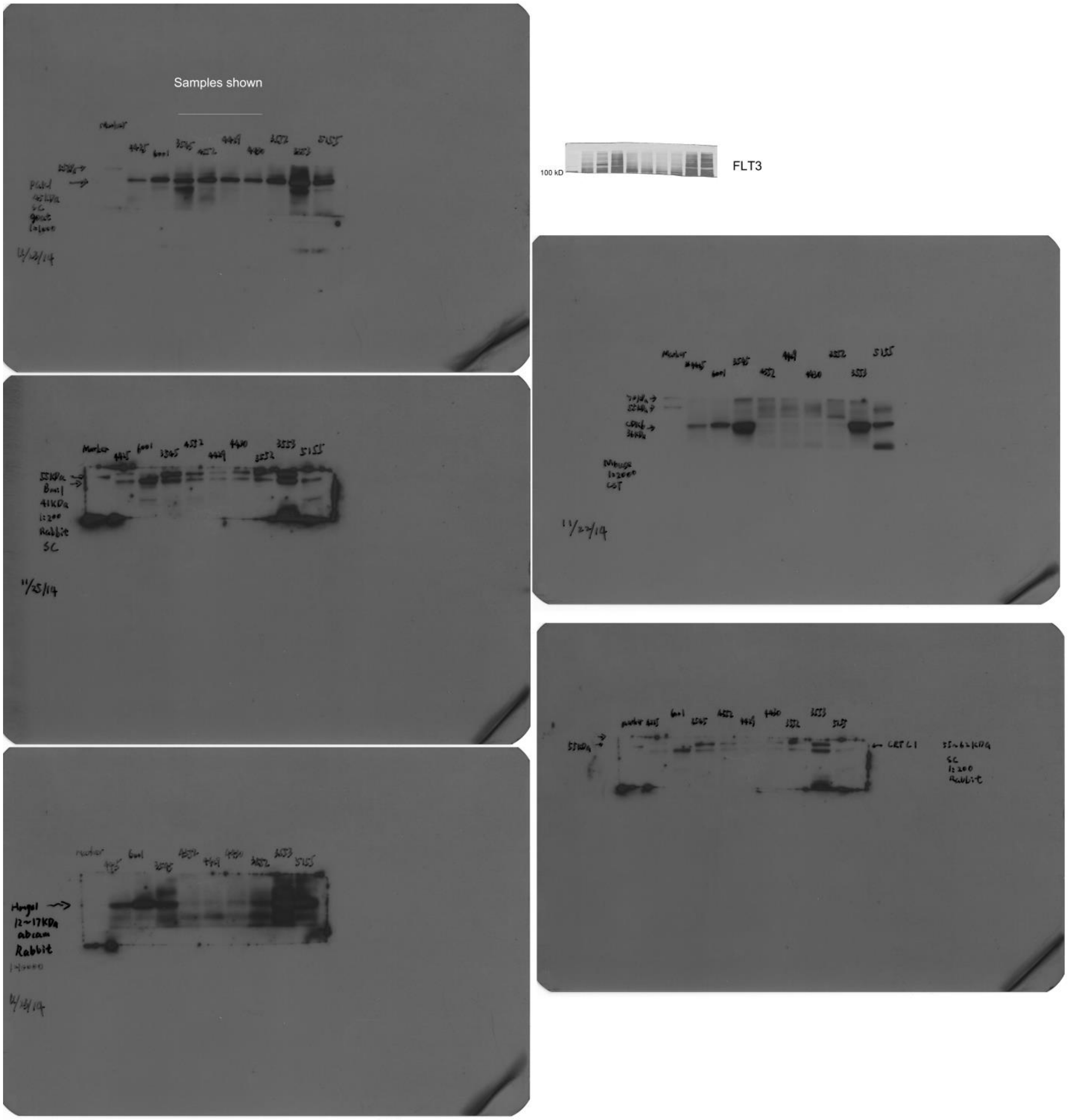

Supplementary Figure 8. Original scans of Western blotting results shown as Supplementary Figure 3c.

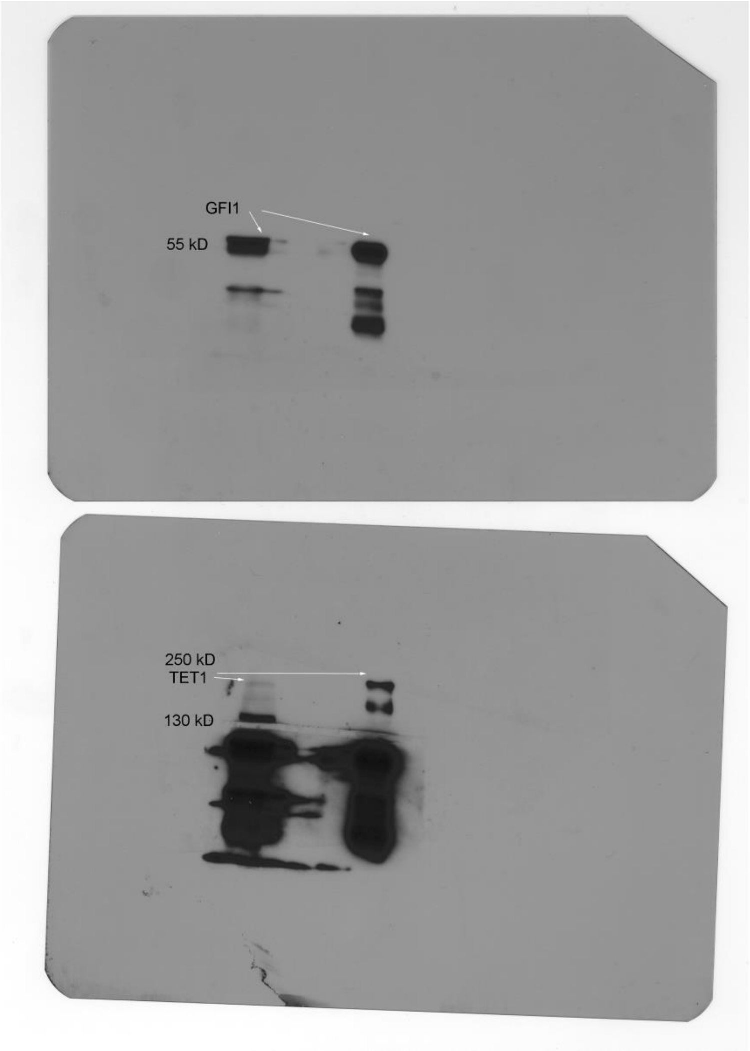

Supplementary Figure 9. Original scans of Western blotting results shown as Figure 6c.

Supplementary Table 1. List of the 22 candidate target genes of miR-22 in AML

Gene	In-house_81S (Pearson correlation analysis of candidate targets and miR-22 in expression)		TCGA_177S (Pearson correlation analysis of candidate targets and miR-22 in expression)		15-mouse-sample set (9 MLL-AF9 AML vs. 6 normal control samples; SAM)		109-human-sample set (100 AML vs. 9 normal control samples; SAM)	
	r	*p*	*r*	*p*	Fold change	*q*-value	Fold change	*q*-value
ANKRD28	-0.43	0.0001	-0.34	<0.0001	1.72	<0.0001	1.67	0.0015
ANKRD52	-0.22	0.0458	-0.17	0.0199	1.22	<0.0001	1.13	0.0348
B3GNTL1	-0.41	0.0001	-0.49	<0.0001	1.28	<0.0001	1.34	0.0107
CDK6	-0.27	0.0139	-0.34	<0.0001	2.38	<0.0001	1.15	0.0131
CRTC1	-0.31	0.0045	-0.16	0.0359	1.32	<0.0001	1.18	0.0173
DDX51	-0.36	0.0010	-0.30	0.0001	1.65	<0.0001	1.18	0.0112
DFFB	-0.26	0.0185	-0.19	0.0103	1.35	<0.0001	1.19	0.0213
ETV6	-0.31	0.0042	-0.40	<0.0001	1.70	<0.0001	1.53	0.0044
FLT3	-0.21	0.0307	-0.35	<0.0001	4.48	<0.0001	2.12	0.0028
MBD3	-0.28	0.0126	-0.21	0.0048	1.31	<0.0001	1.15	0.0238
METTL10	-0.30	0.0066	-0.20	0.0075	1.21	0.0053	1.32	0.0091
MFSD10	-0.46	<0.0001	-0.28	0.0002	1.60	<0.0001	1.61	0.0036
MTL5	-0.29	0.0081	-0.29	0.0001	1.50	<0.0001	1.17	0.0407
MYCBP	NA	NA	-0.15	0.0481	1.11	0.034	NA	NA
OLA1	-0.31	0.0048	-0.35	<0.0001	1.37	<0.0001	1.10	0.0446
RCC2	-0.24	0.0323	-0.23	0.0026	1.37	0.0002	1.33	0.0041
SGTA	-0.34	0.0019	-0.20	0.0082	1.13	0.0014	1.53	<0.0001
SPATS2	-0.33	0.0024	-0.30	0.0001	1.05	0.052	1.21	0.0152
TRIM13	-0.23	0.0392	-0.31	<0.0001	2.58	<0.0001	1.46	0.0016
TRIM46	-0.23	0.0387	-0.26	0.0006	1.25	<0.0001	1.27	0.0054

ZCCHC3	-0.26	0.0187	-0.28	0.0001	1.63	0.0002	1.13	0.026
ZNF512B	-0.39	0.0003	-0.42	<0.0001	2.03	<0.0001	1.38	0.0019

Note: *r*, correlation coefficient; *p*, *p*-value; NA, not available. SAM, significant analysis of microarrays [1]. Notably, *MYCBP* was not included in Affymetrix GeneChip® Human Exon ST Arrays and thus it was absent in our in-house human AML datasets [2,3]; as a result, *MYCBP* was not shown in the original 21 candidate target gene list, although *MYCBP* exhibited a significant inverse correlation in expression with miR-22 in the TCGA dataset and was significantly up-regulated in the mouse AML dataset.

Supplementary Table 2. List of functionally important downstream target genes of the CREB or MYC signaling pathways that exhibit a significant inverse correlation of expression with miR-22 in AML

**Signaling Pathway**	**Gene**	In-house_81S (Pearson correlation analysis of candidate genes and miR-22 in expression)		TCGA_177S (Pearson correlation analysis of canddiate genes and miR-22 in expression)	
		r	*p*	*r*	*p*
**CREB**	*CDK6*	-0.27	0.0139	-0.34	<0.0001
	HOXA7	-0.29	0.0079	-0.16	0.0345
	RGS2	0.39	0.0004	0.50	<0.0001
**MYC**	*BMI1*	-0.24	0.0335	-0.12	0.0455
	FASN	-0.29	0.0091	-0.26	0.0004
	HMGA1	-0.31	0.0051	-0.18	0.0145

Note: *r*, correlation coefficient; *p*, *p*-value.

Supplementary Table 3. Correlation between miR-22 and *TET1/2/3* or *GFI1* in expression in three AML patient cohorts*

AML set	Gene	*r*	*p*
**In-house_81S (n=81)**	***TET1***	**-0.328**	0.0014
	TET2	**0.239**	0.016
	TET3	**0.013**	0.45
	GFI1	**-0.429**	<0.0001
**TCGA_183S (n=183)**	***TET1***	**-0.380**	<0.0001
	TET2	**0.356**	<0.0001
	TET3	**0.327**	<0.0001
	GFI1	**-0.249**	0.0007
**GSE37642_562S (n=562)**	***TET1***	**-0.395**	<0.0001
	TET2	**0.140**	0.0009
	TET3	**0.294**	<0.0001
	GFI1	**-0.262**	0.0005

*Pearson Correlation analysis of *TET1/2/3* or *GFI1* with mature miR-22 (for In-house_81S) or precursor miR-22 (for TCGA_183S and GSE37642_562S) in expression was performed. *r*, correlation coefficient; *p*, *p*-value.

Supplementary Table 4. Effects of G7-miR-22-nanoparticles on mouse blood cell differentiation.

	4 W		8 W		12 W		16 W		20 W		Normal range
	PBS	miR-22	PBS	miR-22	PBS	miR-22	PBS	miR-22	PBS	miR-22	
**WBC (K/µl)**	12.26	11.92	12.06	8.92	4.14	4.18	8.71	9.18	5.74	6.7	1.8-10.7
**NE (K/µl)**	2.79	2.04	2.33	1.85	0.57	0.06	1.16	0.61	0.68	0.88	0.1-2.4
**LY (K/µl)**	8.3	9.48	8.87	6.59	3.38	3.97	6.95	7.97	4.67	5.24	0.9-9.3
**MO (K/µl)**	0.43	0.3	0.5	0.46	0.12	0.14	0.36	0.29	0.33	0.44	0.0-0.4
**EO (K/µl)**	0.57	0.08	0.25	0.02	0.05	0	0.19	0.23	0.05	0.1	0.0-0.2
**BA (K/µl)**	0.16	0.01	0.11	0.01	0.01	0	0.06	0.09	0.01	0.03	0.0-0.2
**RBC (M/µl)**	11.68	10.74	12.2	10.57	9.41	11.78	11.51	12.35	7.71	9.41	6.36-9.42
**PLT (K/µl)**	728	875	847	1215	1253	1246	1007	1354	1070	873	592-2972

Note: Shown are data collected at the indicated time points (i.e. 4, 8, 12, 16 and 20 weeks) post single *i.v.* injection of 0.5 mg/kg of G7-miR-22-nanoparticles (miR-22) or PBS control (PBS). WBC = white blood cells; NE = neutrophils; LY = lymphocytes; MO = monocytes; EO = eosinophils; BA = basophils; RBC = red blood cells; PLT = platelets.

SUPPLEMENTARY REFERENCES

1. Tusher, V.G., Tibshirani, R. & Chu, G. Significance analysis of microarrays applied to the ionizing radiation response. *Proc Natl Acad Sci USA* **98**, 5116-5121 (2001).
2. He, C., *et al.* Young intragenic miRNAs are less coexpressed with host genes than old ones: implications of miRNA-host gene coevolution. *Nucleic Acids Res* **40**, 4002-4012 (2012).
3. Huang, H., *et al.* TET1 plays an essential oncogenic role in MLL-rearranged leukemia. *Proc Natl Acad Sci U S A* **110**, 11994-11999 (2013).
